# B cell receptor isotypes differentially associate with cell signaling, kinetics, and outcome in chronic lymphocytic leukemia

**DOI:** 10.1172/JCI149308

**Published:** 2022-01-18

**Authors:** Andrea N. Mazzarello, Eva Gentner-Göbel, Marcus Dühren-von Minden, Tatyana N. Tarasenko, Antonella Nicolò, Gerardo Ferrer, Stefano Vergani, Yun Liu, Davide Bagnara, Kanti R. Rai, Jan A. Burger, Peter J. McGuire, Palash C. Maity, Hassan Jumaa, Nicholas Chiorazzi

**Affiliations:** 1 Karches Center for Oncology Research, The Feinstein Institutes for Medical Research, Northwell Health, Manhasset, New York, USA.; 2 Institute for Immunology, University Hospital Ulm, Ulm, Germany.; 3 Metabolism, Infection and Immunity Section, National Human Genome Research Institute, NIH, Bethesda, Maryland, USA.; 4 Department of Experimental Medicine, University of Genoa, Genoa, Italy.; 5 Department of Leukemia, MD Anderson Cancer Center, Houston, Texas, USA.

**Keywords:** Immunology, Oncology, Immunoglobulins, Leukemias, Signal transduction

## Abstract

In chronic lymphocytic leukemia (CLL), the B cell receptor (BCR) plays a critical role in disease development and progression, as indicated by the therapeutic efficacy of drugs blocking BCR signaling. However, the mechanism(s) underlying BCR responsiveness are not completely defined. Selective engagement of membrane IgM or IgD on CLL cells, each coexpressed by more than 90% of cases, leads to distinct signaling events. Since both IgM and IgD carry the same antigen-binding domains, the divergent actions of the receptors are attributed to differences in immunoglobulin (Ig) structure or the outcome of signal transduction. We showed that IgM, not IgD, level and organization associated with CLL-cell birth rate and the type and consequences of BCR signaling in humans and mice. The latter IgM-driven effects were abrogated when BCR signaling was inhibited. Collectively, these studies demonstrated a critical, selective role for IgM in BCR signaling and B cell fate decisions, possibly opening new avenues for CLL therapy.

## Graphical abstract

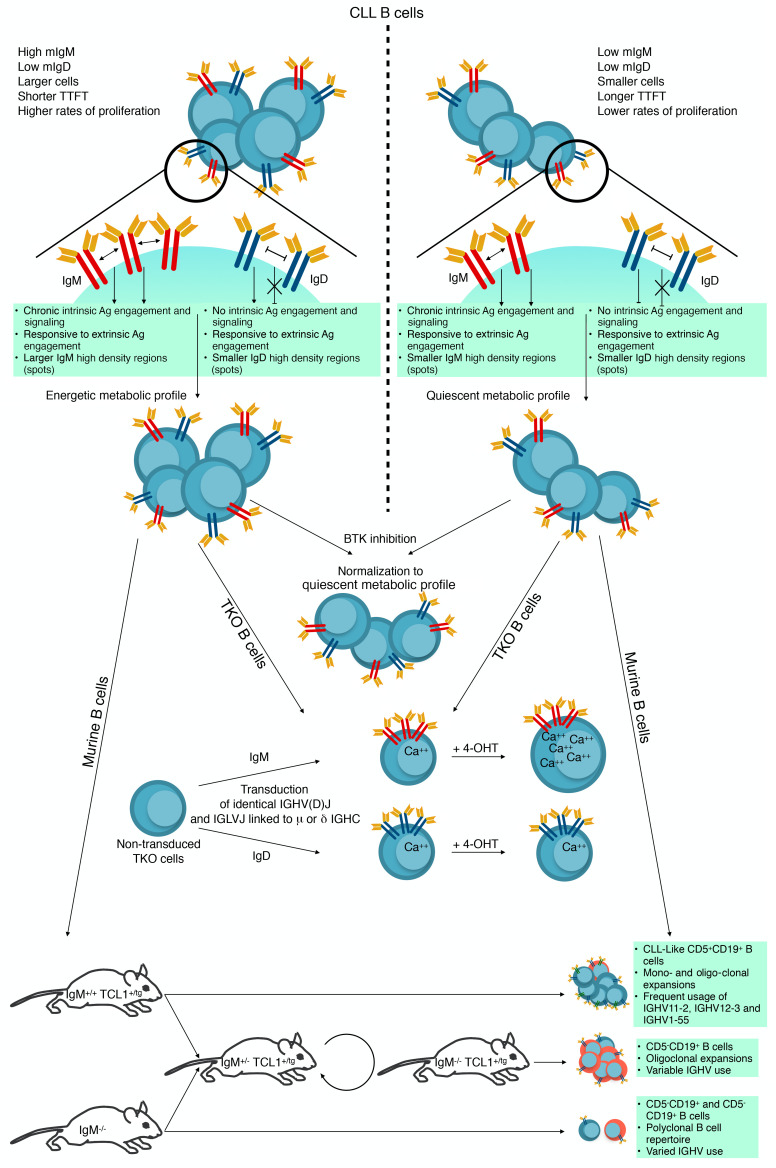

## Introduction

Chronic lymphocytic leukemia (CLL), the most prevalent leukemia among individuals of European descent, is a clonal disease of human B lymphocytes expressing membrane CD5 (1, 2). The disease is highly heterogeneous, with some patients surviving for decades without needing therapy and others requiring therapy shortly after diagnosis due to rapid disease progression. In addition to this heterogeneity between individual patients, CLL also varies intraclonally, containing quiescent and proliferating or recently divided B cell subpopulations (3), and the daily rate at which newly divided cells are generated is linked to disease outcome (4).

Consistent with CLL being a disease of B lymphocytes, the structure and antigen-binding properties of the immunoglobulins (Igs) on the leukemia cell membrane are fundamental for disease development, evolution, and response to therapy (5). In this regard, patients can be divided into 2 groups based on the level of somatic hypermutation in the heavy-chain variable region (IGHV) of the membrane Ig (6). This categorization associates strongly with clinical outcome, as patients with IGHV-mutated CLL (M-CLL) have more indolent outcomes than those with IGHV-unmutated CLL (U-CLL) (7, 8). Moreover, CLL cells exhibit skewed IGHV gene usage (6), and these IGHV genes can associate with specific IGHD and IGHJ genes (9–11) so that approximately 33% of all CLL cases express B cell receptors (BCRs) with remarkable amino acid similarities (12); such rearrangements are referred to as stereotyped BCRs. Moreover, CLL patients displaying the same stereotyped BCRs often share genetic abnormalities (13) and clinical features and courses (14).

Additionally, CLL Igs bind a diverse spectrum of antigens, spanning foreign and autologous molecules (15, 16). It is believed that interactions of such (auto)antigens with membrane-bound Igs (mIgs) (referred to as “BCRs” going forward) on CLL clones generate signals leading to leukemic B cell survival and growth or death (5). This concept is supported by the major beneficial effects for patients when BCR signaling is inhibited (17). Notably, human CLL B lymphocytes and murine B cells made to express polyreactive Igs can also signal through BCRs autonomously, i.e., without a requirement for interaction with an extrinsic antigen (18, 19). This occurs as a consequence of CLL Igs binding themselves, a type of autoreactivity that leads to homotypic interaction (20).

Despite the accumulated knowledge regarding the unique features and clinical importance of the BCR in CLL, much less is known about the mechanism(s) whereby BCRs influence disease development and progression. For example, although most CLL B cells express both IgM and IgD, the physiologic relevance of the 2 isotypes is unclear. Indeed, since the IgM and IgD BCR isotypes bind the same antigen and the loss of function does not drastically perturb B cell development (21, 22), the latter isotype has been considered redundant. Nevertheless, other findings suggest that both isotypes fulfill separate functions. For instance, the relative level and chronology of expression of IgM and IgD during different B cell developmental stages are conserved in mice and humans. Moreover, IgM and IgD are structurally distinct, which can affect Ig function, e.g., the long, flexible hinge region of IgD influences antigen-binding properties, distinguishing that from IgM (23, 24). Additionally, on resting B cells, IgM and IgD reside in independent membrane clusters (23, 25), and their proximities to membrane coreceptors differ and undergo profound reorganization upon activation (23, 26); together, these modulate the quality of BCR signaling and B cell fate (23). Support for the existence of differential functions for the 2 isotypes in CLL derives from the observation that IgM membrane levels have prognostic relevance (27) and engaging these independently can lead to distinct signaling consequences (28).

Using samples from CLL patients whose in vivo leukemia cell birth rates were directly measured (29, 30), we investigated the 2 Ig isotypes on CLL cells, focusing on specific membrane features, clonal metabolic activity, and the involvement of BCR signaling of the autonomous type. We demonstrate that IgM, and not IgD, membrane levels and organization are directly linked with in vivo CLL-cell birth rate and indirectly with cell size and metabolic activity, and that only IgM BCRs mediate autonomous signaling. Consistent with this, we show that the inability to express IgM abolishes the development and expansion of CLL-like B cells in the TCL1 mouse model of CLL. Collectively, these findings define basic mechanisms and regulation of constitutive BCR signaling that indicate a unique role for IgM BCRs in influencing CLL B cell biology and fate.

## Results

### IgM and IgD differ in membrane levels and association with clinical course.

Previous studies differ in the correlation between membrane IgM levels and clinical course (27, 31). So, herein, we addressed this issue and also questioned whether the coexpressed IgD isotype was comparable to IgM in this regard. Using flow cytometry, we measured the levels of the 2 BCR isotypes, defined by mean fluorescence intensity (MFI), on 65 randomly chosen CLL patients of the M-CLL (*n =* 44) and U-CLL (*n =* 21) types (Supplemental Table 1; supplemental material available online with this article; https://doi.org/10.1172/JCI149308DS1). In agreement with D’Avola et al. (27), we found overall levels of IgM significantly higher in U-CLL than M-CLL samples (Supplemental Figure 1A). We also observed that IgD levels were higher in U-CLL cases. However, in contrast to IgM, IgD levels were more variable (Supplemental Figure 1A). This was validated using bivariate analysis (Supplemental Figure 1B).

**Figure 1:**
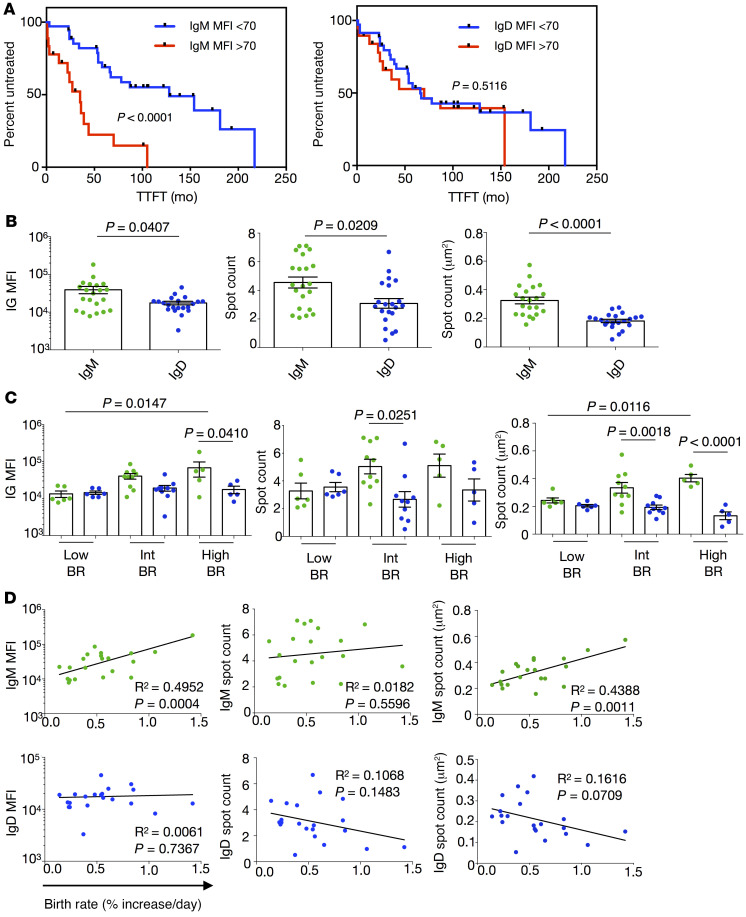
IgM and IgD are differentially expressed and organized on CLL B lymphocyte membranes and correlate with patient clinical course and clonal birth rates in vivo. (**A**) Kaplan-Meier estimates of time to first treatment (TTFT) in CLL patients with IgM or IgD stratified by membrane densities: MFI <70 or >70. Number of cases in the <70 group (IgM, 36; IgD, 36) and in the >70 group (IgM, 19; IgD, 19). (**B**) Comparisons of IgM (green) and IgD (blue) for MFIs, spot counts, and spot areas of all tested samples. (**C**) Comparisons of IgM (green) and IgD (blue) for CLL cases grouped by in vivo BRs (percentage increase in CD19^+^CD5^+^ cells per day): Low (<0.35%), Int (0.35%–0.65%), and High (0.80%–1.42%). Each dot represents the median value of a single patient. Bars represent group means ± SEM. (**D**) Correlation of IgM (green, top) and IgD (blue, bottom) features with matching leukemic B cell BRs for each patient. For statistical analyses, the following tests were applied: (**A**) log-rank, (**B**) Mann-Whitney, (**C**) 1-way ANOVA with Tukey’s test, and (**D**) Pearson’s correlation coefficient.

We also compared time to first treatment (TTFT) of CLL patients grouped by high and low IgM levels based on a receiver-operating-characteristic (ROC) curve, defining an empirical MFI cutoff of 70 (Supplemental Figure 1C). Notably, while IgM^hi^ patients required treatment significantly earlier than IgM^lo^ cases (Figure 1A), IgD^hi^ and IgD^lo^ cases, distinguished by the same MFI cutoff, did not differ in TTFT (Figure 1A). Using the same cutoff for IgD levels did not allow proper separation (Supplemental Figure 1D). Similarly, a cutoff of 50 still allowed grouping patients based on TTFT and IgM but not IgD (Supplemental Figure 1, E and F).

Additionally, the relative roles of the 2 isotypes in TTFT were investigated using an IgD/IgM ratio (DvM ratio). This revealed significantly earlier treatment for patients with DvM of 1.15 or lower, in contrast to those with DvM greater than 1.15 (Supplemental Figure 1, G and H). Increased IgM expression was also detected in ZAP70^+^ CLLs, a subset of patients with enhanced disease aggressiveness (32, 33), whereas IgD was comparable between ZAP70^+^ and ZAP70^–^ (Supplemental Figure 1I). Collectively, these findings are consistent with the levels of IgM being closely link with disease severity (27) and suggest distinct roles for the IgM and IgD isotypes in CLL, as only IgM associates with clinical markers.

### Membrane IgM and IgD organization differ on CLL B lymphocytes and between CLL and normal B cells.

Next, we investigated the membrane characteristics of the 2 Ig isotypes on primary CLL B cells from 2 patient cohorts (Supplemental Table 2), both of whom participated in independent studies quantifying in vivo CLL B cell birth rates (BRs) (29, 30). For both cohorts, leukemic cell BR was directly quantified by measuring the levels of ^2^H incorporated into newly synthesized DNA of CD19^+^CD5^+^ cells (34). Patients in the first cohort were treatment naive but were judged by clinical signs and symptoms to require treatment within 6 months of enrollment. All of these patients subsequently received ibrutinib monotherapy (29). Because of the requirement for treatment, the BRs of most of these clones were high, falling above the clinical cutoff for poor prognosis, which is a 0.35% increase in leukemic cell number per day (BR range, 0.32%–1.42%; Supplemental Table 2 and ref. 30). The second cohort was involved in an independent clinical study not linked to a need for therapy (30); hence, the BRs for most of the CLL cells from this cohort were below the BR cutoff associated with worse clinical course (BR range, 0.14%–0.54%; Supplemental Table 2 and refs. 4, 30). Thus, the use of these 2 cohorts allowed drawing conclusions about the relevance of BR, over the entire clinically relevant span, to BCR-influenced parameters.

For all following experiments with human primary cells, we used imaging flow cytometry (IFC) to study the Ig membrane features of the CLL cells in the 2 cohorts (Supplemental Figure 2, A–G). IFC permits analysis of a large number of individual cells (35), thereby avoiding nonrandom sampling bias imposed by limited cell numbers; it also enables simultaneous analyses of the inherent heterogeneities that could arise due to different degrees of cellular activation and distinct cell division stages within individual CLL clones. Using this approach, we identified B cell populations bearing a CD19^+^CD5^+^ phenotype (Supplemental Figure 2A) and collected single-cell images for each sample (Supplemental Figure 2B). Based on MFI, we evaluated binding specificity of our anti-Igs in comparison to isotype control for CLL B cells in the 2 patient cohorts (Supplemental Figure 2, C and D) and for normal B cells from age-matched, healthy donors (Supplemental Figure 2E). Moreover, for each cell, we determined mean size (Supplemental Figure 2F) and measured the numbers and areas of IgM and IgD high-density regions, i.e., high fluorescence intensity areas on the cell membrane that are hereafter referred to as “spots” (Supplemental Figure 2G).

Intriguingly for CLL clones, median IgM MFI, spot count, and spot area were significantly greater than IgD (Figure 1B). Conversely, healthy donor CD19^+^CD5^–^ B cells displayed comparable IgM and IgD spot counts and spot areas (Supplemental Figure 2H), whereas CD19^+^CD5^+^ B cells had lower IgM than IgD, as determined by MFI (Supplemental Figure 2H). Hence, the measured membrane features differ between IgM and IgD on CLL, and the relative organization of both isotypes differs between CLL and normal B lymphocytes (Figure 1B and Supplemental Figure 2H).

### Membrane IgM, but not IgD, amounts and spot areas associate with CLL B cell birth rates in vivo.

Next, we compared 3 IgM and IgD features (MFI as a measure of amount; spot count; and spot area) among CLL clones grouped based on their leukemic cell BRs, the latter being an indicator of disease aggressiveness (4, 36). Each IgM and IgD feature differed markedly for CLL clones in the higher BR groups (Figure 1C). In contrast, the 2 isotypes were similar in each feature among the low BR cases (Figure 1C). Furthermore, there was a significant, direct linear relationship between IgM MFI and IgM spot area with in vivo leukemic cell BRs (Figure 1D). Similarly, the DvM ratio correlated with in vivo leukemic cell BRs (Supplemental Figure 2I). In contrast, the same parameters for IgD did not show this relationship (Figure 1D).

Together, the above data suggest the density and the topography of IgM associate better with CLL B cell proliferation in vivo than IgD.

Since the density of membrane IgM and IgD defined by MFI might be specious because of differences in the binding abilities of the detecting antibodies used [polyclonal F(ab′)_2_ fragments of goat anti–human IgM and IgD], we measured the absolute number of IgM and IgD BCRs on CLL B cell membranes (Supplemental Figure 3, A–G). Quantum Simply Cellular (QSC) beads (see Supplemental Methods) with different Ab-binding capacities (ABCs) (Supplemental Figure 3A) were incubated with anti-IgM and -IgD mAbs and used to determine the equivalence of MFI and ABCs (Supplemental Figure 3B). The IgM and IgD MFIs of CLL samples stained with the same mAbs were used to calculate the corresponding median ABCs and the equivalent absolute number of BCRs on CLL B cells (see Supplemental Methods for details; ref. 37). In general, CLL B cells expressed a greater absolute number of IgM than IgD BCRs, when quantified individually (Supplemental Figure 3, C and D) and as a group (Supplemental Figure 3E). Also consistent with Figure 1D, a significant direct relationship was observed between IgM BCR numbers and in vivo leukemic cell BRs (Supplemental Figure 3F, left). In contrast, the number of IgD BCRs did not associate with BR (Supplemental Figure 3F, right). Finally, upon human IgM and IgD staining of CLL B cells using F(ab′)_2_ goat polyclonal Abs, the MFIs for both isotypes were plotted with their previously calculated IgM and IgD ABCs, displaying a linear translation with similar comparability (Supplemental Figure 3G). Thus, these results confirm that IgM and IgD densities as measured by the MFI accurately reflect the relative amounts of the 2 isotypes on CLL B cell membranes and confirm that IgM, and not IgD, associates with CLL in vivo kinetics.

### The differential association of IgM and IgD with in vivo leukemic cell BRs is reflected in consequences of BCR signaling.

**Figure 2:**
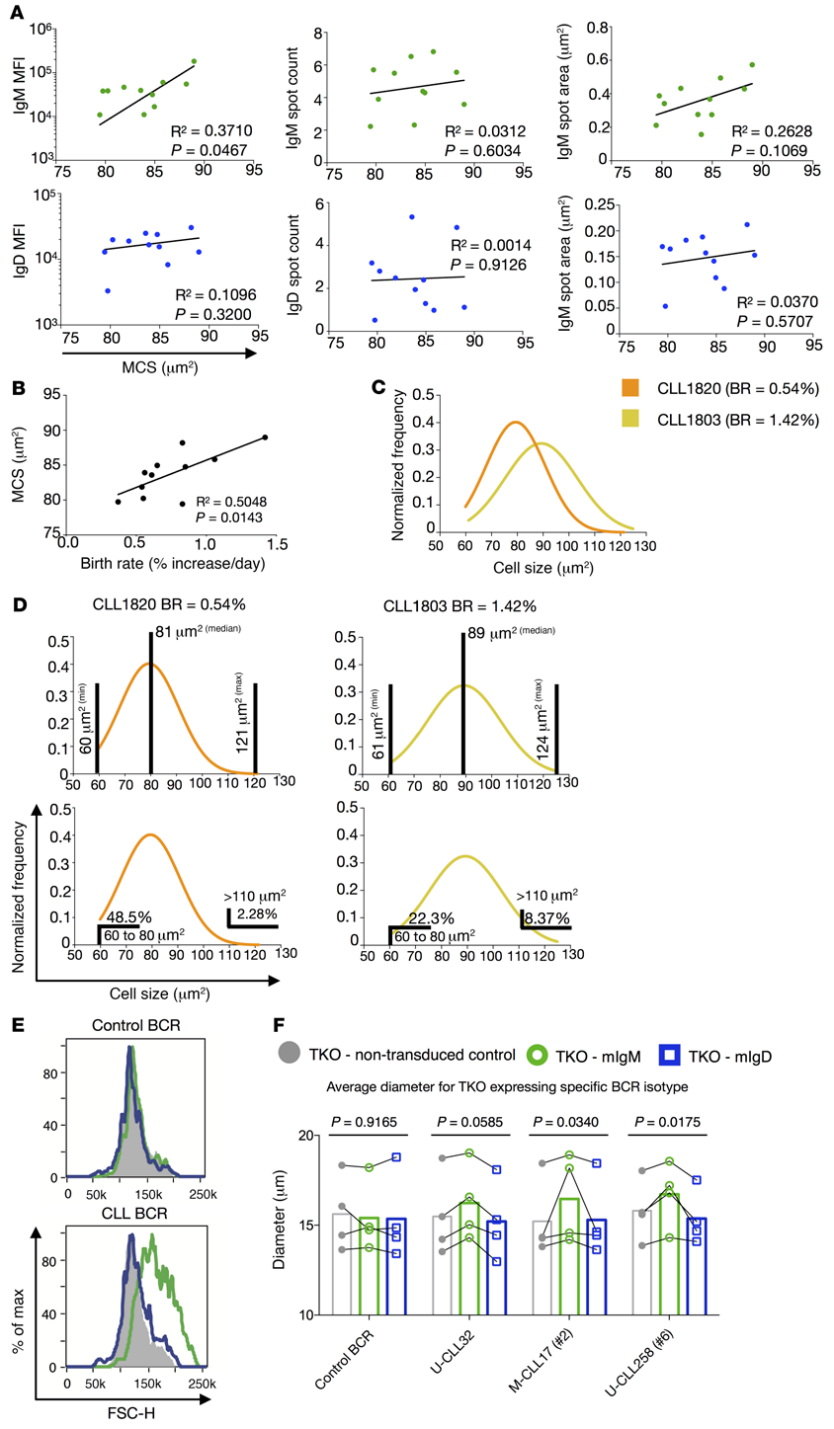
The differential association of IgM and IgD with in vivo CLL birth rate is reflected in median cell size. (**A**) Correlation of IgM (green, top) and IgD (blue, bottom) features with matching median cell size (MCS) in μm^2^. Each dot represents the median value of 1 patient. (**B**) Correlation of MCS with matched patient clonal BRs. (**C**) Comparisons of merged distributions of single-cell areas for CLL1820 (BR 0.54%, orange) and CLL1803 (BR 1.42%, yellow). (**D**) Distribution of single-cell area for CLL1820 (BR 0.54% daily) and CLL1803 (BR 1.42%). Top: Limits and MCS. Bottom: Percentage of small (60–80 μm^2^) and large (>110 μm^2^) cells. The same curves are duplicated at top and bottom to improve graphic display of data. (**E**) Representative FSC-H distribution of TKO cells not transduced (gray), or expressing IgM (green) or IgD (blue) for control BCR (top) and CLL-derived BCR (bottom). (**F**) Average diameter, calculated using SCS standard, for TKO cells nontransduced (gray), or expressing membrane-bound IgM (mIgM, green) or mIgD (blue) for control BCR (top) and CLL-derived BCR (bottom). Four BCRs were tested: (a) control BCR derived from healthy donor CD19^+^CD5^+^ B cells, (b) IGHV-unmutated nonstereotyped CLL BCR, (c) IGHV-mutated stereotyped subset 2 CLL BCR, and (d) IGHV-unmutated stereotyped subset 6 CLL BCR. The following tests were applied for statistical analyses: (**A** and** B**) Pearson’s correlation coefficient and (**F**) Fisher’s test.

Since CLL cells express a membrane phenotype resembling chronically stimulated B cells (38), we asked how cell size and metabolic activity, which change based on cell activation (39, 40), related to IgM and IgD MFIs, spot counts, and spot areas (Supplemental Figure 2F). A significant direct correlation of cell size (measured by the IFC bright-field channel) with IgM MFI and a trend for IgM spot area were identified (Figure 2A). In contrast, IgD MFI, spot count and area, and IgM spot count did not associate with CLL median cell size (MCS) (Figure 2A).

Because our data support a link between IgM (not IgD) and BR (Figure 1, C and D, and Supplemental Figure 3F) and between IgM (not IgD) and MCS (Figure 2A), we questioned if clonal cell size and in vivo BR were linked as well. Indeed, MCS was significantly, directly proportional with BR (Figure 2B), suggesting patients whose leukemic clones proliferate more rapidly have, on average, larger cells. This can be readily appreciated when the single-cell distribution of clones from 2 patients differing in in vivo BRs (CLL1803, 1.42% increase/day versus CLL1820, 0.54% increase/day) were compared (Figure 2, C and D).

Additionally, after grouping the patients into faster and slower BR subgroups, we measured overall MCS as well as the percentage of smallest (60–80 μm^2^) and largest (>110 μm^2^) cells for each case. Notably, neither MCS nor the fraction of the smallest cells differed significantly between the BR subgroups (Supplemental Figure 4A, left and middle), whereas a significantly higher frequency of the largest cells (Supplemental Figure 4A, right) was observed among those cases with faster BRs. The lack of statistical significance for MCS and smallest cells between the 2 BR subgroups is likely due to the progressively increasing cell size in association with BR and/or the number of cases available for study.

Since the percentage of cells in the largest-size fraction associated with the in vivo growth rate of distinct leukemic clones (Supplemental Figure 4A), we surmised that these were cells temporally closer to cell division. Therefore, we measured MCS of the recently divided (CXCR4^dim^CD5^bright^) population (3, 36) in the blood of the same patients. Indeed, the most recently divided cells were markedly larger than the CXCR4^bright^CD5^dim^ cells that had divided earlier (Supplemental Figure 4B). Additionally, the most recently divided cells expressed significantly higher BCR amounts than their older, more quiescent counterparts (Supplemental Figure 4C). Similarly, the intraclonal cells of the largest size displayed significantly higher BCR levels than those with the smallest size (Supplemental Figure 4D). Since bigger cells may present a greater number of mIgs due to their greater membrane surface, we evaluated whether there was an intrinsic higher density of Igs by normalizing the IgM and IgD MFI with the corresponding cellular size. In this instance, only IgM maintained a significant difference among intraclonal subpopulations (Supplemental Figure 4E). Thus, in contrast to IgD, the levels of membrane IgM link with in vivo birth rate and average cellular size measured for the entire clone and for intraclonal members based on time since cell division.

**Figure 3:**
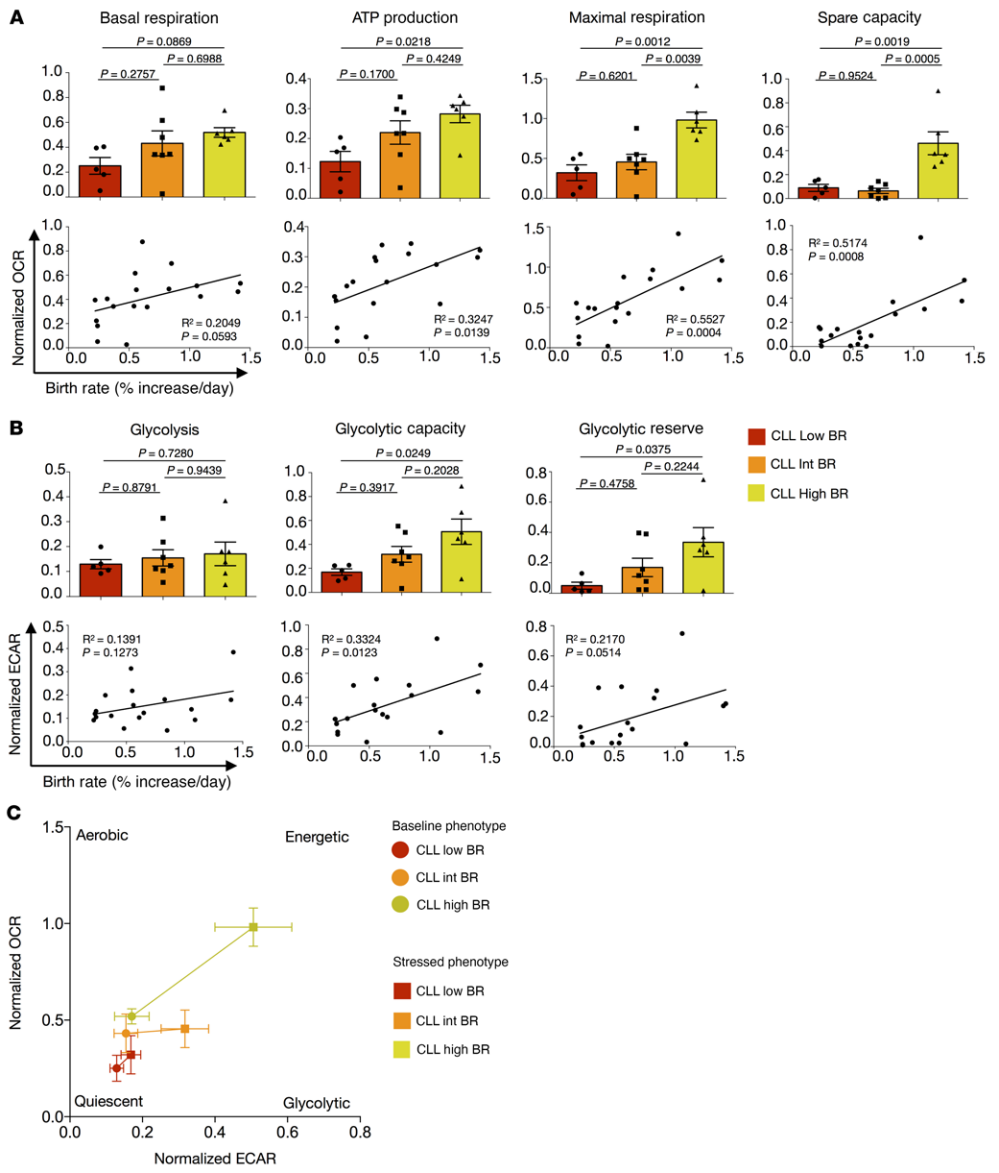
CLL cell metabolic activity associates with CLL B lymphocyte birth rates in vivo. (**A**) Mitochondrial respiration parameters calculated based on oxygen consumption rate (OCR) from mitochondrial stress assay comparing CLL cases grouped by BR (top) or correlating OCR with matched BR for each patient (bottom). (**B**) Glycolytic parameters calculated based on extracellular acidification rate (ECAR) from glycolytic stress assay comparing CLLs grouped by BR (top) or correlating ECAR with matched BR for each patient (bottom). For OCR and ECAR grouped comparisons, bars represent mean ± SEM. Each dot represents the average value of 4 or more replicates for each patient. (**C**) Metabolic profiles for CLL cases grouped by BR. Each dot represents the average OCR and ECAR values for all patients within the group ± SEM. Baseline phenotype = basal respiration (OCR) versus glycolysis (ECAR); stressed phenotype = maximal respiration (OCR) versus glycolytic capacity (ECAR). CLL Low (*n =* 5; BR < 0.35%), CLL Int (*n =* 7; BR = 0.35%–0.65%), and CLL High (*n =* 6; BR = 0.80%–1.42%). OCR and ECAR were normalized, defining 0% as the raw value of 0 and 100% as the last raw value of each data set (or first, whichever was larger), and are presented as fractions. For statistical analyses, 1-way ANOVA with Tukey’s test for bar graphs and Pearson’s coefficient for correlations were used.

Since cell activation also alters cellular metabolic activity (39, 40), we next asked if the in vivo clonal growth rates correlated with metabolic activity in the same patient. To document this directly, we used extracellular flux analysis to compare lower and higher BR CLL clones for differences in oxygen consumption rate (OCR) and extracellular acidification rate (ECAR), measurements of mitochondrial function and glycolytic activity, respectively (Figure 3, Supplemental Figure 5, and ref. 41). Mitochondrial ATP production, maximal respiration, spare capacity (Figure 3A), and glycolytic capacity (Figure 3B) indicated that CLL clones with higher BRs were significantly more metabolically active. Similarly, a trend for basal respiration (Figure 3A) and glycolic reserve (Figure 3B) being associated with higher BRs supported the above conclusion. In contrast, glycolysis did not associate with leukemic cell BR (Figure 3B). Similarly, comparing IgM and IgD MFI with OCR and ECAR measurements highlighted a significant association of IgM with basal respiration and glycolytic capacity and a trend for IgM being linked with mitochondrial ATP production, maximal respiration, and glycolysis (Supplemental Figure 6, A and B). Conversely, IgD displayed only a trend of association with the basal respiration and mitochondrial ATP production (Supplemental Figure 6, A and B).

Additionally, dividing patients into 3 subgroups based on CLL-cell BR (Low, <0.35%; Int, 0.35%–0.65%; and High, 0.80%–1.42%) revealed a similar significant trend when the Low and High BR groups were compared; the Intermediate (Int) group did not differ significantly from the others, consistent with a progressive, linear relationship between BR and metabolic parameters (Figure 3, A and B). This was also reflected in the overall metabolic profile of the 3 subgroups (Figure 3C). Similarly, the IgM^hi^ subgroup reflected a more energetic profile than the IgD^hi^ subgroup (Supplemental Figure 6C). Hence, those CLL clones with higher membrane IgM levels and dividing more rapidly in vivo exhibit increased metabolic activity, marked by an energetic metabolic profile.

### The IgM, but not IgD, isotype drives autonomous BCR signaling and its consequences in vitro.

BCRs on CLL clones can spontaneously self-associate and deliver signals autonomously without binding extrinsic antigen (18), and the BCR structural elements responsible for self-association can differ among CLLs with distinct BCR stereotypes (20). Because our findings indicate that only IgM BCR amounts and aggregation into high-density regions — parameters linked with BCR signaling — associate with in vivo clonal BRs (Figure 1 and Supplemental Figure 3F) and cellular changes associated with BCR signaling (Figures 2 and 3 and Supplemental Figures 4 and 5), we investigated whether autonomous BCR signaling, which is expected to be an ongoing event in CLL clones, differed for the IgM and IgD isotypes. This was done by examining the signaling capacities of 13 CLL-derived BCRs that retained the IGHV-D-J and IGLV-J structures found on the original CLL B cell but expressed either IgM or IgD constant regions (Supplemental Table 3) using triple-knockout (TKO) cells that lack endogenous BCR expression but express 4-hydroxy-tamoxifen–inducible (4-OHT–inducible) ERT2-SLP-65 that enables Ca^++^ influx upon BCR signaling (42).

**Figure 4:**
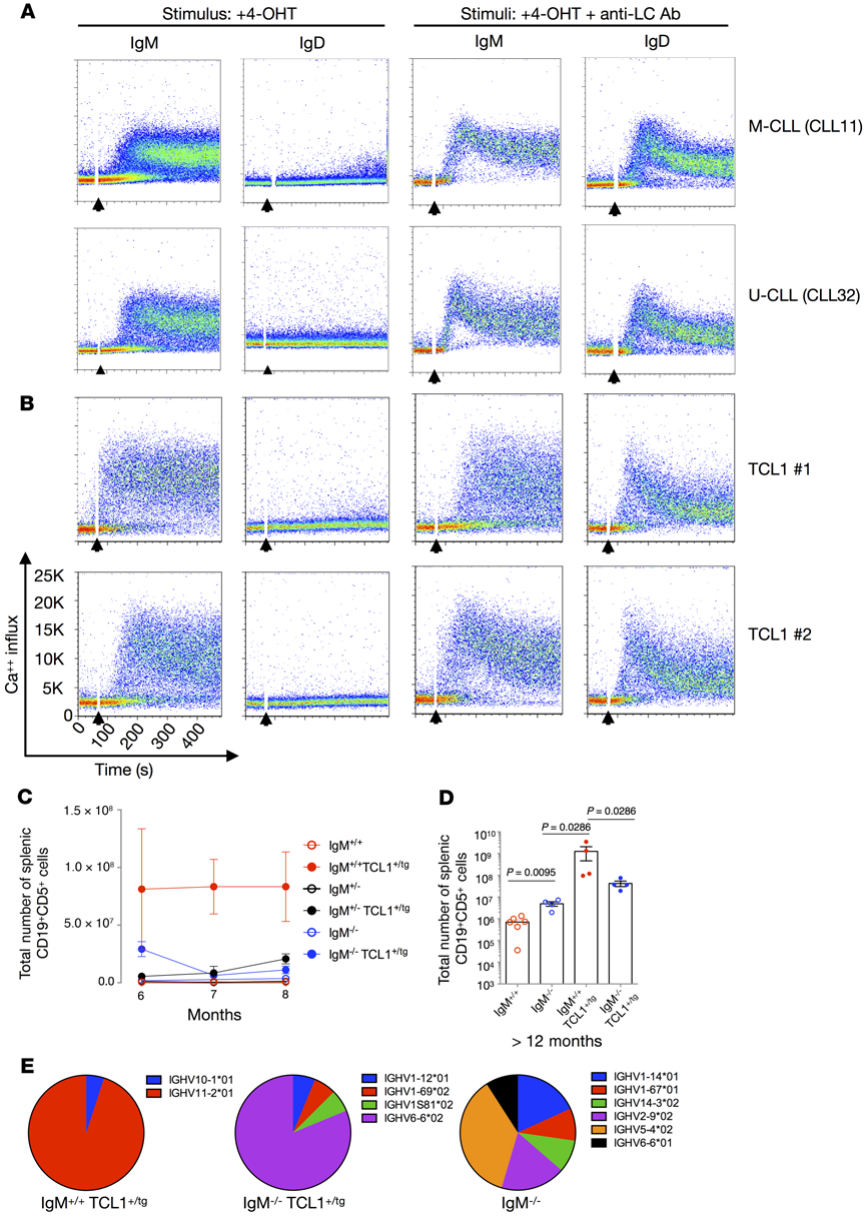
Human and murine CLL IgM and not IgD BCRs mediate autonomous signaling and are required for murine CLL-like disease development. (**A**) Ca^++^ influx analyses of TKO cells expressing IgM or IgD variants of 2 representative CLL-derived BCRs upon stimulation with 4-OHT (left) or 4-OHT plus anti-LC antibody (right). (**B**) IgM or IgD variants derived from TCL1-Tg mice upon stimulation with 4-OHT (left) or 4-OHT plus anti-LC antibody (right). Addition of the stimulus is indicated by black arrows. All CLL BCRs in Supplemental Table 2 (*n =* 17) were tested 3 or more times for autonomous and ligand-dependent calcium mobilization. (**C**) Absolute cell numbers of CD19^+^CD5^+^ cells in spleens from IgM^+/+^ TCL1 (*n =* 3–6), IgM^+/–^ TCL1 (*n =* 3–6), IgM^–/–^ TCL1 (*n =* 3–4), IgM^+/+^ (*n =* 3), IgM^+/–^ (*n =* 3–7), and IgM^–/–^ (*n =* 3) mice. Each point represents median values (±SEM) from mice ranging from 6 to 8 months of age. (**D**) Absolute cell numbers of CD19^+^CD5^+^ cells in spleens from IgM^+/+^ TCL1 (*n =* 4), IgM^–/–^ TCL1 (*n =* 4), and IgM^–/–^ (*n =* 4) mice. Each point represents the number of total CD19^+^CD5^+^ splenic cells of 1 mouse greater than 12 months of age. Bar represents the median number of total CD19^+^CD5^+^ splenic cells within each group. (**E**) Representative samples of the IGHV use frequency from an individual mouse from the IgM^+/+^ TCL1, IgM^–/–^ TCL, and IgM^–/–^ strains.

Upon addition of 4-OHT, BCRs of the IgM isotype induced autonomous signaling, whereas their IgD counterparts did not (Figure 4A), even though they expressed the identical IGHV-D-J and IGLV-D-J rearrangements at equal levels (Supplemental Figure 7A). Notably, both IgM and IgD mediated robust anti–light chain–induced (anti-LC–induced) Ca^++^ influx (Figure 4A), indicating that ligand-dependent BCR signaling was intact for both isotypes. These results were found for M-CLL and U-CLL as well as stereotyped and nonstereotyped CLL BCRs (Supplemental Table 3).

Next, we tested the isotype-specific differences of BCRs from 4 TCL1-transgenic mice that spontaneously develop a CD19^+^CD5^+^ B cell lymphoproliferative disease (43) that resembles aggressive, treatment-resistant human CLL (44). Similar to CLL patients, only the BCRs bearing the IgM isotype led to autonomous signaling (Figure 4B); IgD BCRs failed to do so despite similar levels of expression and a comparable ability to induce Ca^++^ influx upon LC engagement (Figure 4B and Supplemental Figure 7B). Thus, for both human and murine CLL-derived BCRs, only the IgM isotype transmits autonomous BCR signals, even though both can signal when the BCRs are engaged by Abs that mimic extrinsic antigen interaction. These results were qualitatively comparable for all CLL and TCL1 BCRs tested, as reported in Supplemental Table 3.

Finally, an increase in cell size was observed for only those TKO cells expressing IgM but not IgD CLL BCRs. Correspondingly, IgM and IgD BCRs from control CD19^+^CD5^+^ B lymphocytes did not lead to increased cellular size (Figure 2, E and F). Thus, these findings support the observed relationship between IgM levels, BCR signaling, and increased cellular size (Figure 2A).

### IgM is required for the development of a B-1–derived CLL-like leukemia in mice.

To examine the importance of the IgM isotype in vivo, we crossed TCL1 mice with IgM^–/–^ mice (21), and thereafter compared CD19^+^CD5^+^ B cell numbers in the spleen (Figure 4, C and D, and Supplemental Figure 8A) and bone marrow (Supplemental Figure 8B) of offspring at various ages. Within 6 to 8 months, splenic CD19^+^CD5^+^ B cells were elevated only in wild-type IgM^+/+^ animals carrying the TCL1 transgene. None of the homozygous (IgM^–/–^) or heterozygous (IgM^+/–^) KO animals or mice lacking the TCL1 transgene showed this increase (Figure 4C). Correspondingly, IgM^+/+^ TCL1 mice older than 12 months showed accumulation of CD19^+^CD5^+^ B cells in the spleen, compared with age-matched IgM^–/–^ TCL1 and IgM^–/–^ animals (Figure 4D). Similar accumulations of B cells with the same phenotype were found in the bone marrows of IgM^+/+^ TCL1 mice compared with age-matched IgM^–/–^ TCL1 and IgM^–/–^ mice, confirming that IgM expression is necessary for CLL-like lymphoproliferation in the TCL1-transgenic mouse model (Supplemental Figure 8B).

When comparing lymphocyte subpopulations in the spleens, we found decreased numbers of CD19^+^CD5^+^ CLL-like cells and a gain of CD19^+^CD5^–^ B cells in the IgM^–/–^ TCL1 mice (Supplemental Figure 8C). Conversely, IgM^+/+^ and IgM^–/–^ mice lacking TCL1 overexpression did not exhibit a loss of CD19^+^CD5^+^ or CD19^+^CD5^–^ B cell subpopulations (Supplemental Figure 8C). The latter finding rules out the possibility that the absence of IgM downregulates CD5 expression and supports progression to leukemia as a cause for the decreased numbers of CD19^+^CD5^+^ CLL-like cells in the IgM^–/–^ TCL1 animals. However, the gain of CD19^+^CD5^–^ B cells in the IgM^–/–^ TCL1 mice might still represent a phenotypically unusual CLL-like expansion. Normal and systemic lupus erythematosus–like murine B-1 cells and TCL1 CLL-like cells use a very restricted set of *IGHV* genes, mostly *IGHV11-2*, *IGHV11-3*, and *IGHV1-55* (44, 45). Thus, we evaluated *IGHV* usage in splenic B cells of IgM^–/–^ TCL1, IgM^+/+^ TCL1, and IgM^–/–^ mice (Figure 4E and Supplemental Table 4), finding that IgM^+/+^ TCL1 splenic B cells of each mouse exhibited an oligoclonal population that used one (or more) of the reported *IGHV*s. In contrast, IgM^–/–^ TCL1 did not express any of the *IGHV* genes overrepresented in normal and autoimmune B-1 and CLL-like B cells. Finally, IgM^–/–^ mice displayed a polyclonal population and a different IGHV usage with respect to IgM^+/+^ TCL1 as well (Figure 4E and Supplemental Table 4). Thus, the IgM^–/–^ TCL1 CD19^+^CD5^–^ B cells do not resemble IgM^+/+^ TCL1 CD19^+^CD5^+^ expansion both phenotypically (CD5 expression) and for *IGHV* usage. The IgM^–/–^ TCL1 CD19^+^CD5^–^ B cell expansion is likely due to the genetic pressure of TCL1 overexpression, leading to the formation of other lymphoma/leukemia in these mice. This further supports the notion that lack of IgM expression does not allow CLL-like cell development.

Importantly, IgM^–/–^ TCL1 mice possess a CD19^+^CD5^+^IgD^+^ B cell population from which the leukemia could initiate. However, despite the presence of these cells, a CLL-like leukemia did not develop in these animals. Since only IgM BCRs from humans and mice with CLL can signal autonomously (Figure 4, A and B), the lack of CD19^+^CD5^+^IgD^+^ CLL-like cells supports our contention that IgM-specific autonomous signaling is required for the overgrowth and accumulation of leukemic B cells in TCL1 mice. This corroborates the finding that the development of CLL-like B cells in TCL1 mice requires autonomous and antigen-mediated BCR signaling (46).

### Inhibition of BTK by ibrutinib blocks autonomous BCR signaling in vitro and diminishes metabolic activity in CLL cells in vivo.

**Figure 5:**
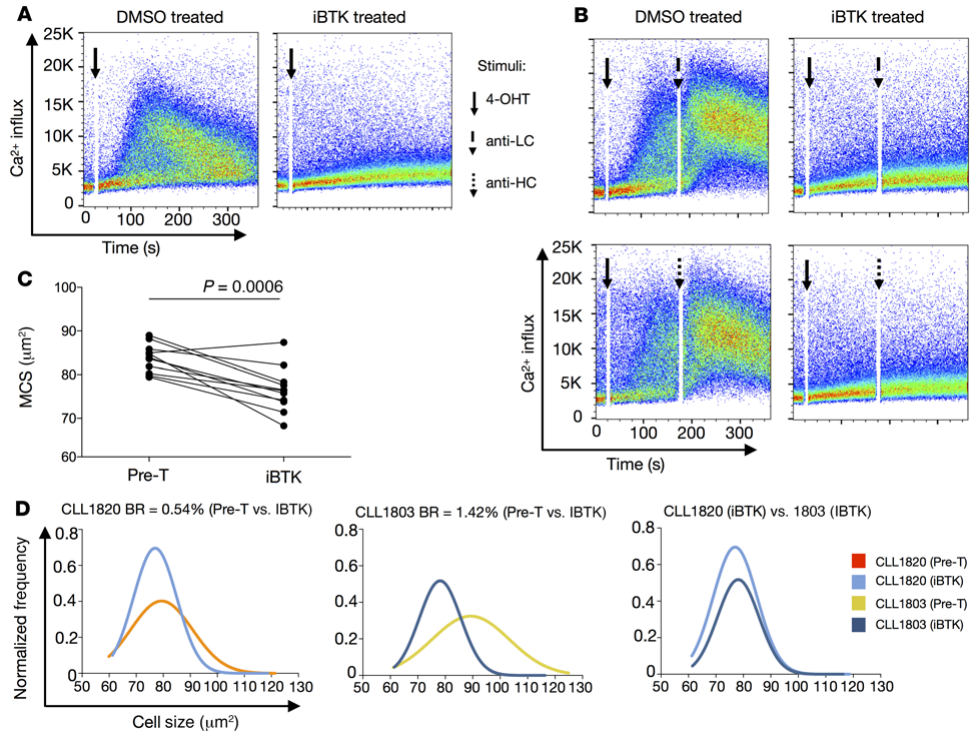
Ibrutinib treatment affects both autonomous and surrogate antigen–induced BCR signaling, thereby reducing MCS. (**A**) Representative Ca^++^ influx analyses of TKO cells expressing IgM CLL–derived BCRs pretreated with ibrutinib (iBTK, right) and solvent (DMSO, left) for autonomous signaling upon stimulation with 4-OHT. (**B**) Ca^++^ influx analyses of TKO cells expressing IgM CLL–derived BCRs pretreated with ibrutinib (iBTK, right) and solvent (DMSO, left) for ligand-dependent signaling using anti-light chain (anti-LC) (top) and anti-heavy chain (anti-HC) (bottom) mAbs upon stimulation with 4-OHT. Addition of the stimulus is indicated by black arrows. Three or more experiments were performed for each CLL BCR (*n =* 4) for autonomous and ligand-dependent signaling in the absence or presence of ibrutinib. (**C**) Median cell size (MCS) before (Pre-T) and during ibrutinib (iBTK) treatment. Each dot represents the median value of an individual patient. (**D**) Distribution of single-cell areas for CLL1820 (BR = 0.54% daily) before (Pre-T, orange) and during (light blue) ibrutinib treatment, and for CLL1803 (BR = 1.42% daily) before (Pre-T, yellow) and during (dark blue) treatment. The Mann-Whitney test was used for these statistical analyses.

Since ibrutinib, an inhibitor of Bruton’s tyrosine kinase (iBTK), effectively blocks BCR signaling in vitro (47) and in patients (48), we assessed the BTK dependency of autonomous signaling using TKO cells expressing CLL IgM BCRs (Figure 5, A and B). Ca^++^ influx was inhibited by ibrutinib pretreatment (Figure 5A), supporting BTK as necessary for autonomous BCR signaling. As expected, ligand-dependent BCR signaling induced by anti-LC and anti–heavy chain (anti-HC) Abs was also inhibited by ibrutinib (Figure 5B).

During in vivo ibrutinib treatment, we did not observe statistically significant changes in Ig features for both IgM and IgD (data not shown). This could be due to the point in the disease at which we tested, at which treatment started, and/or the duration of the treatment. In addition, the role that kinases upstream of BTK play in Ig reorganization is complex and unappreciated. Finally, the number of cases available for study might have been insufficient to define significant effects. Indeed, the changes in IgM and IgD organization are believed to be strongly associated with an “inside-out” Syk activity that modifies actin cytoskeleton and BCR organization upon engagement (26). Thus, inhibition of BTK, a kinase downstream of Syk, may not lead to significant changes in BCR organization, while still influencing the signaling consequences further along in the pathway. However, analyses of leukemic cell size from patients treated with ibrutinib had a strong effect on cell size, significantly reducing MCS in 10 out of 11 patients (Figure 5C). Moreover, the degree of cell size change during ibrutinib therapy was reflected differentially among patients, in that clones with higher in vivo BR cases had the largest cell size reductions. The differential effect is easily appreciated when comparing single-cell areas for the 2 patients differing in in vivo BRs (CLL1803, 1.42% increase/day versus CLL1820, 0.54% increase/day) (Figure 5D and Supplemental Figure 9A). Similarly, when the samples were divided into faster and slower BR subgroups, only the former displayed significant decreases in MCS and downsizing was most evident in the largest (more active) cells (Supplemental Figure 9B). In line with this, the higher BR group also had a greater increase in the numbers of the smallest (less active) cells upon iBTK therapy (Supplemental Figure 9B).

**Figure 6:**
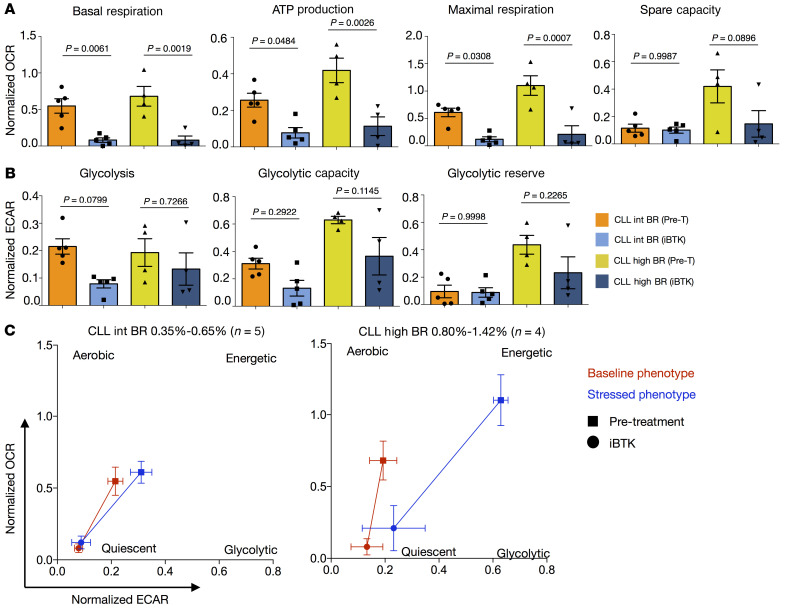
Ibrutinib treatment downregulates metabolic activities associated with membrane IgM levels and in vivo CLL birth rate. (**A**) Mitochondrial respiration parameters calculated based on OCR from mitochondrial stress assay comparing CLL BR Int (*n =* 5; BR = 0.35%–0.65%) and High (*n =* 4; BR = 0.80%–1.42%) before (Pre-T) and during in vivo ibrutinib (iBTK) treatment. (**B**) Glycolytic parameters calculated based on extracellular acidification rate (ECAR) from glycolytic stress assay comparing CLL cases with Int (*n =* 5; BR = 0.35%–0.65%) and High (*n =* 4; BR = 0.80%–1.42%) BRs before (Pre-T) and during in vivo ibrutinib (iBTK) therapy. Bars represent mean ± SEM. Each dot represents the average value of 4 or more replicates for a single patient. (**C**) Changes in metabolic profiles during iBTK treatment. Each dot represents the average OCR and ECAR values for all patients within the BR group ± SEM. Baseline phenotype = basal respiration (OCR) versus glycolysis (ECAR); stressed phenotype = maximal respiration (OCR) versus glycolytic capacity (ECAR). Before (squares) and during treatment (circles) changes are shown for baseline phenotype (red) and stressed phenotype (blue) for CLL clones with Int BR (0.35%–0.65%, left) and High BRs (0.80%–1.42%, right). OCR and ECAR were normalized, defining 0% as the raw value of 0 and 100% as the last raw value of each data set (or first, whichever was larger) and are presented as fractions. For statistical analysis, 1-way ANOVA with Tukey’s test was applied.

In line with this, OCR showed an overall significant reduction in the 2 BR groups for basal respiration, ATP production, and maximal respiration (Figure 6A and Supplemental Figure 9C). Additionally, an inhibitory trend was observed for mitochondrial spare capacity (Figure 6A) and glycolytic parameters (Figure 6B and Supplemental Figure 9D). The depression of metabolic activity during ibrutinib treatment, especially mitochondrial respiration in the higher BR samples, led to a quiescent metabolic profile (Figure 6C).

Thus, inhibition of BCR signaling associates with a regression in cell size and metabolic profile in vivo, and the changes are proportionally greater for the larger and more metabolically active leukemic B cells. Collectively, our findings assign the effects solely to the IgM isotype.

## Discussion

The leukemic B lymphocytes in CLL patients are highly dependent on signals delivered by the BCR for survival and growth. Since IgM and IgD initiate these signals, we studied the amount, distribution, and signal transmission capacity of the 2 isotypes, and then associated these with leukemic cell BRs in patients, a measure of disease activity, and with cell size and metabolic activity, consequences of cell activation and division. A strength of these studies is the use of samples from patients for whom clonal in vivo BRs had been measured. Because some patients’ BRs were measured just prior to starting iBTK monotherapy, we were also able to directly associate our findings before and when BCR signaling was inhibited.

Using this approach, we documented that IgM and IgD BCRs on the same leukemic B cell are organized and function differently. On unmanipulated ex vivo cells, we found that IgM is present in greater amounts and is organized into more and larger high-density regions/spots than for IgD. Since higher clonal BRs directly associate with poor prognosis (4), our finding that IgM expression and to a lesser extent IgM spot area associate with leukemic cell proliferation rates in vivo suggests an explanation as to why CLL patients with clones exhibiting the highest IgM amounts experience the worst clinical outcome (27).

We also found that, despite IgM and IgD bearing identical antigen-binding domains, only IgM molecules mediate autonomous signaling in human and murine CLL B lymphocytes. Thus, in some manner, the IgM constant region leads to or allows Ig-Ig self-association that promotes and appears necessary for autonomous BCR signaling (42). Support for a direct contribution by the Ig constant region comes from the crystal structure of a distinct, stereotyped subset of CLL clones (subset 4) in which the constant region provides an amino acid essential for Ig-Ig self-association (20). In contrast, the crystal structure of another stereotyped subset (subset 2) indicates that self-association can be independent of the IGH constant region (20). Thus for some CLL cases, indirect mechanistic models are necessary to explain a selective role for IGH in self-association and resultant autonomous signaling. In this regard, the IgM and IgD isotypes exhibit distinct thresholds of activation due to differences in hinge-region length and flexibility that could affect self-association, as it does for binding to antigens differing in epitope valency (24). Moreover, since each set of CLL clones appears to require a structurally distinct internal epitope to allow Ig-Ig self-association (20), both possibilities could occur.

We also discovered further evidence for IgM membrane amounts having a primary role in the development of leukemia by using TCL1 mice, a model of CLL, in which expression of the IgM constant region was not possible (21). In this setting, autonomous signaling and leukemia development did not occur, even though CD19^+^CD5^+^IgD^+^ B cells existed in these animals, suggesting that IgM expression is involved in the biased use of the specific IGHVs in B cells. Indeed, TCL1 mice expressing IgM mainly displayed *IGHV11-2*, *IGHV12-3*, and *IGHV1-55*, which are the most commonly used IGHVs in the restricted repertoires exhibited by normal and autoimmune B-1 cells and by CLL-like murine CD5^+^ B cells (44, 45). In contrast, IgM^–/–^ TCL1 mice did not express any of these B-1–associated IGHV genes.

Collectively, these findings indicate that, even though IgM and IgD on the same B cell clone display identical antigen-binding regions, only IgM delivers autonomous signals to CLL cells to promote clonal sustenance in patients and mice. This is in line with studies in TCL1 mice demonstrating a requirement for autonomous signaling in the genesis of CLL (46).

This conclusion is supported by the finding that IgM BCR amounts and in vivo CLL-cell BRs correlate with enhanced cellular metabolic activity, most of which is accounted for by changes in oxidative phosphorylation (OXPHOS). BCR signaling and OXPHOS are directly linked in CLL, as evidenced by genetic ablation studies knocking out PI3Kδ (49), as well as by our findings with iBTK treatment in patients showing that regression of metabolic activity from an energetic to a quiescent state associates with the level of disease activity. Our data concerning the ability of ibrutinib to modulate mitochondrial metabolism are also supported by a recent publication addressing markers of poor prognosis in CLL (50). Overall, enhanced OXPHOS is a predictor of poor outcomes in CLL (49), and our studies link IgM signaling to this critical metabolic pathway.

Thus, key issues are how to meld the relationships between (a)increased IgM BCR levels and assembly into more frequent and larger high-density regions/spots, (b) faster in vivo BRs and enhanced cell size and heightened metabolic activity, and (c) increased IgM BCR levels and faster in vivo BRs with the types of BCR signaling. Regarding the latter, in vivo iBTK therapy, which we showed blocks autonomous as well as extrinsic antigen–mediated BCR signaling, led to significant decreases in cell size and clonal metabolic activity that were proportional with in vivo BRs. While inhibition of autonomous signaling by ibrutinib might be expected, this is the first formal report to our knowledge of such an action by ibrutinib and, thus, has implications for our understanding of CLL biology as well as how the drug might act as a therapeutic agent. These findings indicate that clonal metabolic activity is significantly influenced by cellular activation through the BCR, consistent with the activated membrane phenotype of CLL cells (51) and the expression of Cdk4 and cyclin E indicating positioning at the early G_1_ phase of the cell cycle (52). Moreover, the findings are in line with the metabolic phenotype of BCR-triggered normal B cells (39, 53). Lastly, this possibility is also supported indirectly by the discovery that CLL BCRs form oligomers autonomously, in contrast to normal B cells (54).

Finally, it is crucial to understand the relative contributions of autonomous BCR signaling, mediated by self-association induced by binding to antigens intrinsic to the BCR, and classical BCR signaling induced by Ig binding to antigens extrinsic to the BCR, to the findings reported here and to link these to CLL B cell biology. Regarding autonomous signaling, the structures that allow this are inherent to IgM. Consequently, IgM BCRs always have the opportunity to self-associate and autonomously signal, regardless of the location in the body of an individual cell at any point in time. For classical BCR signaling, since both IgM and IgD engage the same extrinsic antigen, the 2 isotypes together initiate this form of signaling. However, in order to bind Ig-extrinsic antigens and thereby lead to cell stimulation and growth (as opposed to anergy), leukemic B cells should reside in solid tissues, e.g., lymph nodes (LNs) (55–57), where a greater avidity of antigen-Ab interactions can occur due to the immobilization of the target antigens. This is consistent with the documentation that CLL B cell proliferation occurs more extensively in LNs (57). Since not all cells in the leukemic clone can simultaneously interact with extrinsic antigen in the correct anatomic location, changes in membrane topography, cell size, and metabolic activity induced by classical BCR signaling would be manifested in only a small subset of cells.

Thus, we propose the following chronologic scenario. CLL cell proliferation is initiated by engaging BCR-extrinsic antigen(s) (auto or foreign antigens) via IgM/IgD BCRs in proliferation centers of the stromal microenvironment (57), and this involves only the relatively small subpopulation of leukemic cells at those sites (3). These cells undergo metabolic reprogramming to meet the energetic demands of cell growth (58). When these recently divided cells exit tissue niches and arrive in the circulation, autonomous IgM-mediated BCR signaling maintains them in an augmented metabolic state that enhances survival. Consequently, autonomous signaling allows some of these cells to survive long enough to traffic back to and reenter tissue niches where they receive prosurvival signals, mediated by classical BCR signaling. Thus, autonomous signaling provides survival signals that permit survival until the leukemic cells reach the tissue microenvironment. In contrast, signaling initiated by BCR-extrinsic antigens drives proliferation of those cells at an appropriate anatomic location where they interact with clonally relevant antigens. This process varies for cells based on their rates of growth since the relative proportion of CLL cells that have divided is greater for those with faster BRs.

Although this proposal stresses a role for autonomous signaling in the periphery, such stimulation would also occur centrally and likely alter responses and cell fates initiated by classical BCR signaling. In this regard, since productive IgD engagement requires multivalent antigens (59), which are more likely available as immobilized substrates in secondary lymphoid tissues (55, 57), IgD-mediated signaling could rescue anergic cells in the circulation, the latter possibly induced by IgM BCRs (60). This is in line with IL-4 increasing IgM expression and reenergizing the BCR signaling capacities of CLL cells (61, 62). Thus, in this model, the influences of the 2 BCR isotypes would differ at different phases of the CLL life cycle. Further studies of the interaction of these 2 types of BCR signaling and their effects on maintaining and supporting survival and expansion of CLL cells in distinct anatomic niches are needed to test this hypothesis.

## Methods

Further information can be found in Supplemental Methods.

### Study design.

This study was designed to compare the membrane topography of the clonally restricted IgM and IgD BCRs on CLL cells and to assess the contributions their respective signaling played in the development and progression of the disease.

In a cohort of 65 randomly selected patients (Supplemental Table 1), we confirmed that IgM BCR amounts correlated with the previously described need for earlier treatment (27). To investigate this association more precisely and to relate it to leukemic cell in vivo BR and metabolic activity, we used cryopreserved samples from 2 cohorts of untreated patients (*n =* 12 and 11, respectively; Supplemental Table 2) that differed in clonal BRs. In general, Cohort 1 consisted of patients with faster BRs and Cohort 2 with slower BRs. The first group received iBTK therapy within 6 months of determining the in vivo CLL cell kinetics; for the second group there was no such requirement. For both groups, BRs were measured directly by determining the levels of deuterium (^2^H) incorporated into newly synthesized DNA of leukemic cells replicating in vivo after the patients had drunk deuterated water (^2^H_2_O). Additional information about the 2 clinical trials and in vivo kinetics of these patients is available in Burger et al. and Messmer et al. (29, 30). Because patients with defined in vivo leukemic cell BRs who received treatment with ibrutinib therapy within 6 months of this measurement are extremely scarce, the choice of samples and their employment for each assay was not blind and was based on material availability (Supplemental Table 2). For these patients, the time points for the samples analyzed were preselected as those taken before and 4 weeks after initiation of iBTK treatment. Samples taken before treatment for Cohort 1 and those of Cohort 2 were used to investigate the association of in vivo BR with membrane IgM and IgD features and clonal metabolic activity profile. IgM and IgD membrane features included cell-surface density (MFI) and aggregation in high-density regions (spots). As ibrutinib inhibits BTK activity and intracellular BCR signaling, samples from Cohort 1 taken during treatment were used to determine whether IgM and IgD BCRs, clonal metabolic activity measured by cell size using IFC, mitochondrial respiration and glycolysis activity using Seahorse technology, and in vivo BRs were intimately related to BCR signaling.

The abilities of IgM and IgD BCRs to transduce intracellular signaling was tested using the established TKO cell line approach (42), by expressing CLL-derived IGHV-D-J and IGLV-J rearrangements as IgM or IgD. CLL Ig rearrangements were arbitrarily chosen to cover the skewed and yet heterogeneous range found in patients, thus including those falling into the U-CLL and M-CLL subgroups and into stereotyped and nonstereotyped subgroups (6, 14). Direct proof for the importance of Ig isotype in the development and progression of CLL in vivo was confirmed by crossing TCL1 mice (43), a widely used murine model of aggressive CLL (44), with IgM^–/–^ animals that, from early B cell development, lack μ HC expression but retain δ HCs (21).

### Patient sample.

Cryopreserved samples from untreated CLL patients with active disease who previously drank ^2^H_2_O to determine CLL cell BRs in vivo and who subsequently received treatment with only ibrutinib were used. These are referred to as Cohort 1. A second, independent cohort of samples whose in vivo kinetics were determined but did not necessarily require treatment was also used. Detailed data about the 2 clinical trials and in vivo kinetics of these patients are publicly available (29, 30).

### Animal model.

TCL1-transgenic mice (43, 44) were crossed with IgM^–/–^ mice (21) to obtain the following genotypes: IgM^+/+^TCL1^+/tg^, IgM^+/–^TCL1^+/tg^, and IgM^–/–^TCL1^+/tg^. Mice were sacrificed at 6, 7, 8, and >12 months of age, respectively. Mice of control strains (IgM^+/+^, IgM^+/–^, and IgM^–/–^) were sacrificed at the same time points.

### Statistics.

For statistical analysis, GraphPad Prism software was used. The statistical significance (*P* value) of patient TTFT was calculated using the log-rank test. For mouse populations and cell-size grouped analyses, Mann-Whitney test was used. Wilcoxon’s rank test was used for paired analysis (Ig clusters and cell size before and during treatment). Correlations were calculated with Pearson’s coefficient and lines fit with linear regression analysis. For multiple comparisons, 1-way ANOVA with Tukey’s correction was used. Statistical significance was defined by a *P* value of less than 0.05 (2-tailed). Data are presented as mean ± SEM, unless otherwise indicated.

### Study approval.

Human studies were carried out in accordance with the Declaration of Helsinki and approved by the Institutional Review Boards of The Feinstein Institute for Medical Research and Freiburg University. All patients provided written informed consent. Animal experiments were performed according to institutional ethical allowance and in compliance with the guidelines of the German law, license number 1288, regional board Tübingen, Germany, and approved by the Institutional Animal Ethics Committee of Ulm University under permission number 1288.

## Author contributions

ANM, EGG, MDVM, PCM, PJM, HJ, and NC conceptualized the study. ANM, EGG, and MDVM developed the methodology. ANM, EGG, MDVM, and PCM performed validation studies, conducted formal analyses of the data, and prepared the figures. ANM, EGG, MDVM, PCM, TNT, AN, GF, SV, YL, and DB carried out the investigation. KRR, JAB, PJM, HJ, and NC provided resources. ANM, MDVM, HJ, and NC wrote the original draft of the manuscript. ANM, EGG, PCM, PJM, HJ, and NC reviewed and edited the manuscript. HJ and NC supervised the study. ANM, EGG, HJ, and NC provided project administration. HJ and NC acquired funding.

## Supplementary Material

Supplemental data

Supplemental table 1

Supplemental table 2

Supplemental table 3

Supplemental table 4

## Version 1 11/23/2021

In-Press Preview

## Version 2 01/18/2022

Electronic publication
