# Supplementary material for: B cell receptor isotypes differentially associate with cell signaling, kinetics, and outcome in chronic lymphocytic leukemia
Source: J Clin Invest. 2022 Jan 18;132(2):e149308. doi: 10.1172/JCI149308 (PMC8759784; doi:10.1172/JCI149308)
Supplement: Supplemental data [file jci-132-149308-s066.pdf]

Supplementary Information

Supplementary Materials and Methods

Animal Models. TCL1 transgenic mice were crossed with IgM knockout mice to obtain the following genotypes: $IgM^{+/+}$ $TCL1^{+/tg}$, $IgM^{+/-}TCL1^{+/tg}$, $IgM^{-/-}TCL1^{+/tg}$(1-3). The control genotypes included $IgM^{+/+}$, $IgM^{+/-}$ and $IgM^{-/-}$. Mice were euthanized using CO_2 at 6, 7, and 8 months of age when B cell clonal expansion are usually obvious. Homozygous strains ($IgM^{+/+}$ $TCL1^{+/tg}$, $IgM^{-/-}$ $TCL1^{+/tg}$, $IgM^{+/+}$ and $IgM^{-/-}$) were followed and euthanized also at later time points (> 12 months), when most mice exhibit CLL like disease. At sacrifice, spleens were harvested, and single cell suspensions made by homogenizing the organ, filtering through a 45 μm filter. To study bone marrow (BM) cells, femurs and tibiae were removed and crushed in a mortar with containing PBS + 2% FBS, filtered through a 45 μm mesh to remove particulate debris and to obtain single cell suspensions. Blood (20-50 μl) was collected with a Heparin-wetted (Heparin-Natrium 25000 I.E. Ratiopharm, #N68542.04) needle from the tail vein for flow cytometric analysis. Red blood cells (RBCs) in the cell suspensions from all organs were lysed by resuspending the cell pellet in 1 ml of Qiagen RB-lysis solution (#158904) and incubating the sample for 5 min at RT. RBC lysis of blood was done by adding 1 ml of Qiagen RB-lysis solution directly to whole blood, followed by an incubation step for 5 min at RT. This was done twice. RBC lysed single cell suspensions were used for further analyses, such as flow cytometry. Animal experiments were performed according to institutional ethical allowance and in compliance with the guidelines of the German law, license no. 1288, regional board Tübingen, Germany.

Flow cytometry. To evaluate membrane IgM and IgD levels on murine BM and splenic cells, as well as human CLL samples from the randomly selected patient cohort used in Figure 1 and S1, single cell suspensions were stained with fluorescent-labeled antibodies. Murine samples were incubated with anti-mouse Igϰ-APC-Cy7 (goat polyclonal, Southern Biotech); anti-mouse IgM-FITC (goat polyclonal, Southern Biotech); anti-mouse IgD-PE (clone 11-26c, Southern Biotech); anti-mouse CD19-APC (clone 1D3, BD Biosciences); anti-mouse CD5-PE-Cy7 (clone 53-7.3, eBioscience). CLL samples were stained with anti-human ϰ or λ L chain antibodies conjugated with biotin (polyclonal, Southern Biotech) and detected with either Streptavidin-PE-Cy7 or Streptavidin-eFluor 450; anti-human IgM-PE (goat polyclonal, Southern Biotech); anti-human IgD-PE (goat polyclonal, Southern Biotech). Cells were treated with Fc Block anti-mouse CD16/32 (clone 2.4G2, BD Bioscience) or human Fc Block (BD Bioscience) prior to staining, and then analyzed by FACS analysis using LSR Fortessa (BD Biosciences).

Measurement of membrane IgM and IgD BCRs on CLL B cells. The number of membrane IgM and IgD BCRs were determined by FACS analysis. For data normalization and standardization, Quantum Simply Cellular beads (QSC, Bangs Laboratories, Inc.) were used according to the manufacturer's instructions.

For CLL sample staining, $2x10^6$ thawed PBMCs were washed twice with PBS and incubated with 50μL of FACS buffer containing either Alexa Fluor 647-labeled monoclonal mouse anti-human IgM (cat. # 314536; Biolegend) or anti-human IgD (cat. # 348228; Biolegend) together with anti-human CD5 PE-Cy7 (cat. # 364008; Biolegend) and anti-human CD19 Alexa Fluor 488 (cat. # 302219; Biolegend). Saturation staining conditions for anti-IgM and anti-IgD antibodies were optimized by standard titration, applying 2-fold increasing doses until a <5% gain of MFI occurred. Final doses for monoclonal anti-IgM and anti-IgD were 2 μg/mL for $2x10^6$ cells in 100μL FACS

Buffer. Thereafter, IgM and IgD MFIs of $CD19^{+}CD5^{+}$ CLL B cells were normalized over their respective isotype controls and used to calculate the median numbers of ABCs. The ABC equivalent was translated to the number of BCR as described (4). CLL B cells were stained in parallel with polyclonal goat anti-human IgM PE (cat. # 2020-09; Southern Biotech) and anti-human IgD PE (cat. # 2030-09; Southern Biotech) with a saturation point of 2.5 μg/mL for $2x10^{6}$ cells in 100μL FACS Buffer. IgM and IgD MFIs of $CD19^{+}CD5^{+}$ CLL cells were normalized over their respective isotype controls and plotted versus their corresponding known ABCs revealing a comparable linear translation for both isotypes.

Use of Triple Knock-Out (TKO) cells to test autonomous and ligand-mediated BCR signaling. TKO cells were transduced with retrovirus as described (5, 6). Briefly, IG H and L chains were expressed using the biomolecular fluorescence complementation vector system (5). CLL-derived BCRs were amplified from patient samples. Human *IGHV-IGHD-IGHJ* rearrangements were fused to the μ or δ human constant regions by PCR, whereas the complete human L chains were used. For virus production, Phoenix cells were transfected using FuGeneHD according to the manufacturer's protocol. Supernatants were collected 48 h after transfection and used for transduction of TKO cells (5, 6).

Measurement of intracellular calcium mobilization was performed as described (7). Briefly, 1×10^{6} cells per sample were incubated with 5 μg/ml of Indo-1 AM (Invitrogen) in Iscove's Basal Medium (IBM; Biochrom GmbH) supplemented with 1% heat-inactivated FCS (cat. # P30-3302, Lot P170804; PAN Biotech) and Pluronic F-127 (Invitrogen) for 45 minutes at 37°C. Afterwards, cells were pelleted and resuspended in IBM with 1% FCS for measurement. To characterize the BCR signaling capacity, ERT2-SLP65 function was induced by stimulating with 1 µM 4-

hydroxytamoxifen (4-OHT; Sigma Aldrich) after acquiring baseline tracings for 30 s. For ligand-dependent stimulation of BCR signaling, 5 µg/ml anti-BCR was used in addition to 4-OHT. Calcium influx was measured using LSR Fortessa (BD Biosciences). For cell size measurement, FACS analyses were performed and the FSC-H values, normalized with size calibration standard (SCS) beads ranging from 4-11 μm diameter according to the manufacturer's instructions (Bangs Laboratories, Inc.), were compared. TKO cells, expressing empty vectors or IgM or IgD BCRs derived from CLL B cells or normal B cells from healthy controls, were incubated with Sytox blue live-dead dye (Invitrogen), and analyzed by FACS in parallel with similarly treated SCS beads. Upon exclusion of dead cells, FSC-H values were plotted against the SCS bead standard to obtain the median cell diameter. For signaling inhibition experiments, ibrutinib (final dose 5 µM) or vehicle alone from a 5 mM stock in DMSO was added to TKO cells 16-18 hours before measurement.

Extracellular flux analysis. Oxygen consumption rate (OCR) and extracellular acidification rate (ECAR) were determined using the Seahorse XF Cell Mito Stress and Seahorse XF Glycolysis Stress Test kits and measured using a Seahorse XF96 analyzer (Seahorse Bioscience). Patients PBMC were defrosted, resuspended at a concentration $4x10^5$ live cells/well in XF assay media (Seahorse Bioscience) with 1 mM sodium pyruvate and with or without 10 mM glucose for Mitochondrial and Glycolysis stress kit, respectively, and attached to wells with Cell-Tak (Corning) according to manufacturer's instructions. Final concentrations of compounds upon injection were: 0.5 µM Oligomycin, 1 µM FCCP, 0.5 µM Rotenone + antimycin A (AA) for Mitochondrial stress test, and 10mM Glucose, 1 µM Oligomycin, 50 mM 2-Deoxy-d-glucose (2-DG) for Glycolysis stress test. OCR and ECAR were calculated and recorded by the Seahorse XF-96 software. The average value of the three recordings before and after each injection was used to

calculate the metabolic parameters as follows. Mitochondrial stress kit was used for: a) Basal Respiration = OCR before oligomycin minus OCR after Rotenone & antimycin A; b) Proton Leak = OCR after oligomycin minus OCR after Rotenone & antimycin; c) ATP Production = Basal Respiration minus Proton Leak; d) Maximal respiration = OCR after FCCP minus OCR after Rotenone & antimycin A; e) Spare Capacity = Maximal respiration minus Basal Respiration. Glycolysis stress kit was used for: a) Glycolysis = ECAR after glucose minus ECAR after 2-DG; b) Glycolytic Capacity = ECAR after oligomycin minus ECAR after 2-DG; c) Glycolytic Reserve = Glycolytic Capacity minus Glycolysis. OCR and ECAR were normalized by 0% for the raw value of 0 and 100% as the last raw value of each dataset (or first, whichever was larger). Results are presented as fractions.

Cell preparation and staining for Imaging Flow-Cytometry. CLL PBMCs were isolated by density gradient centrifugation with Ficoll-Paque Plus (GE Healthcare) and used after thawing frozen aliquots that had been cryopreserved in liquid nitrogen in 10% DMSO and 90% FCS. To avoid membrane BCR changes, all procedures were carried out on ice and PBS with 0.02% NaN_3 and 1% FCS (Staining Buffer). 100 μl of $2x10^7$ cells/ml were aliquoted in V-shaped 96 well plate and incubated for 30 min with murine anti-human mAbs and/or goat $F(ab')_2$ anti-human as per manufacturer concentrations. Cells were subsequently centrifuged at 300g for 10 min, washed 3 times with 200 μl staining buffer and suspended with 100 μl BD Cytofix solution (BD Bioscience) for 1h before acquisition started.

Antibodies used for Imaging Flow-Cytometry. For isotype-specific BCR cluster analysis, the following antibodies were used: anti-CD5-phycoerythrin (PE) (clone UCHT2) and anti-CD19-Pacific Blue (clone HIB19) were from Biolegend; goat $F(ab')_2$ anti-IgM-FITC, goat $F(ab')_2$ anti-IgD-FITC and goat $F(ab')_2$ IgG isotype control were from Southern Biotech.

Imaging Flow-Cytometry Acquisition and Analysis. Cells from the patient cohort that consumed ^2H_2O were automatically imaged in flow using an Amnis ImageStream X MKII (EMD Millipore) at 60x magnification. The ImageStream X captures simultaneously up to 12 images/cell through 12 channels (ch1-12) divided between 2 cameras (6 channels/camera, Ch1-6 for camera 1 and Ch7-12 for camera 2). The channels correspond to brightfield (one per camera, Ch1 and Ch9 respectively), side scatter (SSC), and up to 9 different fluorescence emission spectra. A halogen light source was used for the brightfield images and 4 lasers at 488nm, 405nm, 561nm and 642nm were used for fluorochrome excitation. For this study, up to 7 channels were used as follow: Ch1 = Brightfield; Ch2 = anti-IgM-FITC or anti-IgD-FITC; Ch3 = anti-CD5-PE; Ch7 = anti-CD19-Pacific Blue; Ch12 = SSC. Single color samples were acquired in absence of brightfield and SSC excitation wavelengths and used to create a compensation matrix applied to the experimental samples to correct possible fluorescence spillover among different channels. After compensation, experimental sample images were analyzed by IDEAS software (EMD Millipore). For cellular images analysis, masks attributed to a specific channel (from M01 = Ch1 to M12 = Ch12) were used to differentiate the region of interest (ROI, i.e., cell membrane, fluorescence spot) from the background picture. To identify cells with images in best focus, the brightfield channel was interrogated with the feature *Gradient RMS* resulting in Gradient RMS_M01_Ch1 algorithm. Since cells with better focus have a higher value we excluded all events below a threshold value of 50. Upon exclusion, cells were plotted for their brightfield features *Area* (Area_M01) versus *Aspect Ratio* (Aspect Ratio_M01). Single cells, with an intermediate area value and high aspect ratio, were gated. Finally, the algorithm *Intensity* was used to define $CD19^+CD5^+$ CLL cells. This feature calculates the sum of the pixel intensity units within the chosen mask. Thus, $CD19^+CD5^+$ cells were gated after plotting CD5-PE (Intensity_MC_Ch03) versus CD19-Pacific Blue

(Intensity_MC_Ch7). Accordingly, $CD19^+CD5^+$ cells were gated after plotting CD5-PE (Intensity_MC_Ch03) versus CD19-Pacific Blue (Intensity_MC_Ch7). Hence, all further analyses were based on Image Focus > Single Cells > $CD19^+CD5^+$ cells gating strategy. For single cell area or "cell size", Ch1 images were used with the software embedded feature *Area* (that calculates the mask area in μm^2) and a *Morphology* mask that defines as ROI the entire cell resulting in the following combined algorithm: Area_Morphology (M01, Ch01). IgM- and IgD-BCR spot counts (number of clusters per cell) were based on the Ch2 images using a *Spot* mask that arranges the ROI around bright detail fluorescence (FITC) intensity with a radius of 3 or less pixels. The *Spot Count* feature was implemented to calculate the number of fluorescent spots resulting in the algorithm: Spot Count_Spot (M02, Ch2, Bright). Similarly, the average area of the spots (average cluster size per cell) was obtained by applying the *Area* feature to the same M02 mask used for spot count, calculating the total area of the ROI. Then, the area value was divided for the spot count value to obtain the mean spot area per cells in μm^2 result in the combined algorithm: [Area_Spot (M02, Ch2, Bright)]/[Spot Count_Spot (M02, Ch2, Bright)].

Supplementary References

1. Bichi R, et al. Human chronic lymphocytic leukemia modeled in mouse by targeted TCL1 expression. *Proc Natl Acad Sci U S A.* 2002;99(10):6955-60.
2. Yan XJ, et al. B cell receptors in TCL1 transgenic mice resemble those of aggressive, treatment-resistant human chronic lymphocytic leukemia. *Proc Natl Acad Sci U S A.* 2006;103(31):11713-8.
3. Lutz C, et al. IgD can largely substitute for loss of IgM function in B cells. *Nature.* 1998;393(6687):797-801.
4. Mattila PK, et al. The actin and tetraspanin networks organize receptor nanoclusters to regulate B cell receptor-mediated signaling. *Immunity.* 2013;38(3):461-74.
5. Meixlsperger S, et al. Conventional Light Chains Inhibit the Autonomous Signaling Capacity of the B Cell Receptor. *Immunity.* 2007;26(3):323.
6. Su YW, and Jumaa H. LAT links the pre-BCR to calcium signaling. *Immunity.* 2003;19(2):295-305.
7. Storch B, et al. The Ig-alpha ITAM is required for efficient differentiation but not proliferation of pre-B cells. *Eur J Immunol.* 2007;37(1):252-60.

Supplementary Figures and Legends

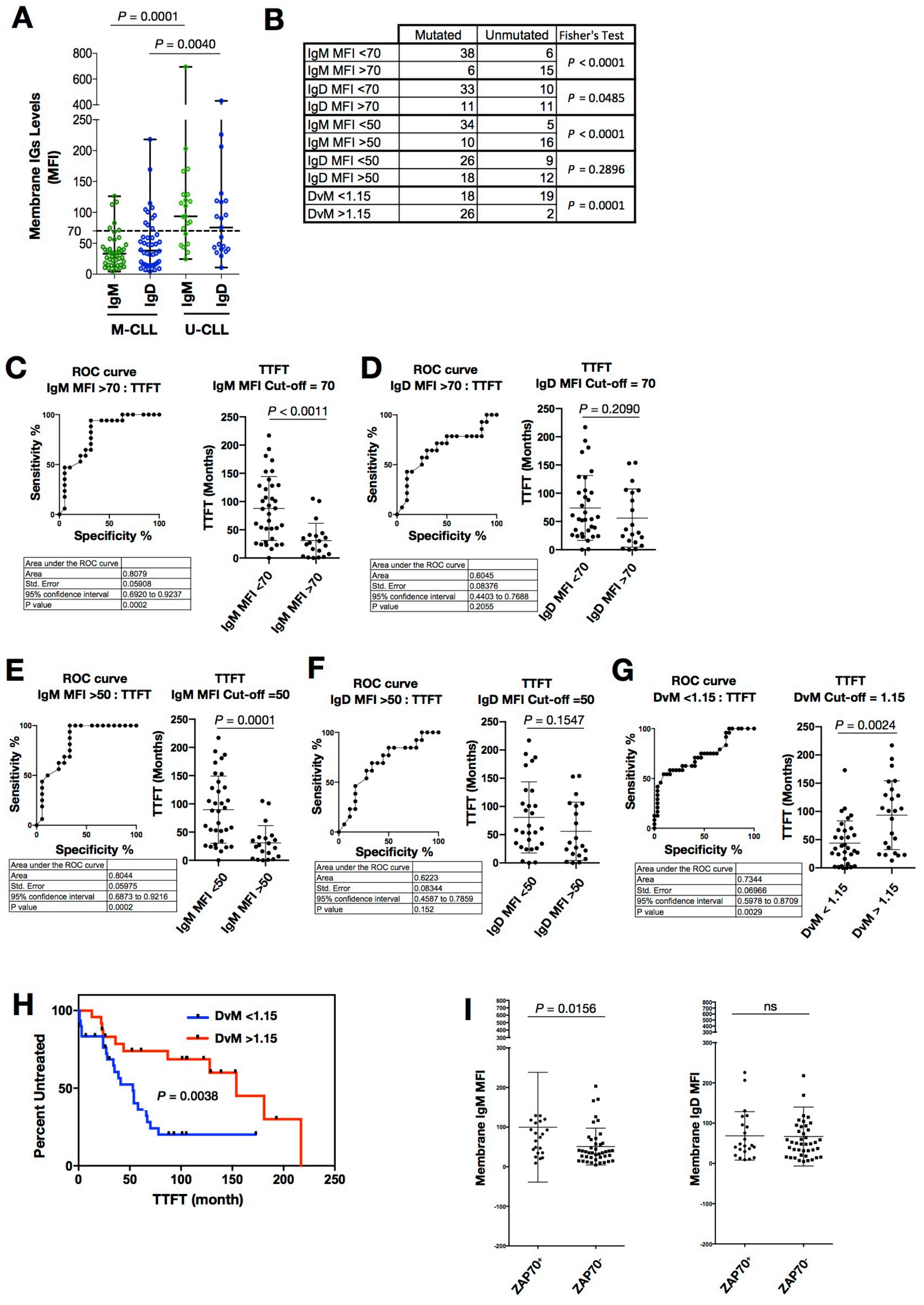

Figure S1. Correlation of IgM and IgD membrane levels with prognostic markers. A. Mean fluorescence intensities (MFIs) of membrane IgM and IgD were determined for 45 M-CLL and 22

U-CLL samples. Each symbol represents one patient. Dashed line at 70 MFI is an empirically-defined cut-off for IgM MFI below which 66% of CLL patients cluster. **B.** Evaluation of the association of IgM and IgD MFI and IgD/IgM ratio (DvM) cut-offs with IGHV mutation status. The Contingency Table shows the number of cases belonging to M-CLL and U-CLL subgroups based on the IGs MFI cut-off. Fisher's exact test was used to calculate the association between the two variables. **C.** ROC for IgM MFI of 70 (left) and TTFT upon division of CLL based on IgM MFI of 70 (right). **D.** ROC for IgD MFI of 70 (left) and TTFT upon division of CLL based on IgD MFI of 70 (right). **E.** ROC for IgM MFI of 50 (left) and TTFT upon division of CLL based on IgM MFI of 50 (right). **F.** ROC for IgD MFI of 50 (left) and TTFT upon division of CLL based on IgM MFI of 50 (right). **G.** ROC for relative expression ratio of IgD versus IgM (DvM) with empirically defined cut-off of DvM = 1.15 (left) and TTFT upon division of CLL in DvM <1.15 and >1.15 groups (right). **H.** Kaplan-Meier estimates of time to first treatment (TTFT) in CLL patients stratified by DvM ratio < 1.15 or > 1.15. Number of cases in the < 1.15 group (n = 37) and in the > 1.15 group (n = 28). **I.** Comparisons of IgM MFI (left) and IgD MFI (right) for $ZAP70^+$ versus $ZAP70^-$ cases. For statistical analyses, (**A-G and I)** the Mann-Whitney test was applied and, **(H)** the Log-rank test was used.

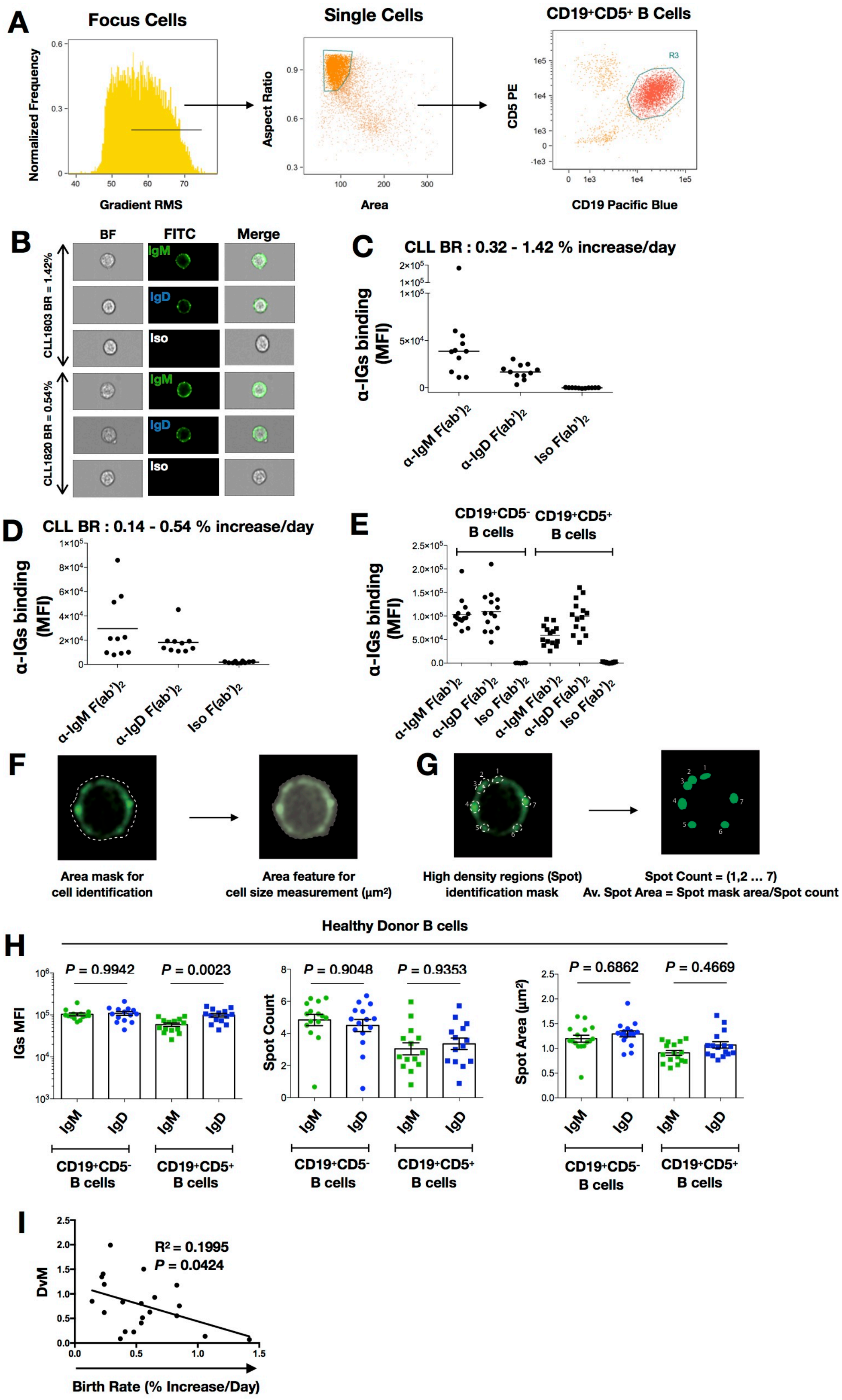
A
Focus Cells
Normalized Frequency
Gradient RMS
Single Cells
Aspect Ratio
Area
CD19+CD5+ B Cells
R3
CD5 PE
CD19 Pacific Blue
B
BF
FITC
Merge
IgM
IgD
Iso
CLL1803 BR = 1.42%
CLL1820 BR = 0.54%
C
CLL BR : 0.32 - 1.42 % increase/day
α-IGs binding (MFI)
α-IgM F(ab')2
α-IgD F(ab')2
Iso F(ab')2
D
CLL BR : 0.14 - 0.54 % increase/day
α-IGs binding (MFI)
α-IgM F(ab')2
α-IgD F(ab')2
Iso F(ab')2
E
CD19+CD5- B cells
CD19+CD5+ B cells
α-IGs binding (MFI)
α-IgM F(ab')2
α-IgD F(ab')2
Iso F(ab')2
α-IgM F(ab')2
α-IgD F(ab')2
Iso F(ab')2
F
Area mask for cell identification
Area feature for cell size measurement (μm2)
G
High density regions (Spot) identification mask
Spot Count = (1,2 ... 7)
Av. Spot Area = Spot mask area/Spot count
Healthy Donor B cells
H
P = 0.9942
P = 0.0023
IGs MFI
P = 0.9048
P = 0.9353
Spot Count
P = 0.6862
P = 0.4669
Spot Area (μm2)
IgM
IgD
IgM
IgD
CD19+CD5- B cells
CD19+CD5+ B cells
I
DvM
R2 = 0.1995
P = 0.0424
Birth Rate (% Increase/Day)

Figure S2. IgM and IgD fluorescence detection and analysis of high density regions ("spots") and cellular size. A. Progressive gating strategy of focus (left), single cells (middle), and $CD19^+CD5^+$ B cells (right) using Imaging Flow Cytometry. Using the IDEAS software, focused images of cells were selected based on "Gradient RMS" values, a feature that evaluates image focus quality measuring difference in pixel values. To select single cells, the feature "Aspect Ratio", which defines the ratio of the axes for the cell within the image, was used in association with the feature "Area" that defines the median cell size in μm^2. CLL B cells were further selected as $CD19^+CD5^+$ based on the intensity signal associated with those two markers. **B.** Sample of Imaging Flow Cytometry acquired images for detection of membrane IGs with FITC-conjugated anti-IGs (IgM/IgD) and FITC-conjugated control IG (Iso). Two exemplary samples are displayed: CLL1803 (BR 1.42% daily) and CLL1820 (BR 0.54% daily). ImageStream X Mark II was used for image acquisition using a 60X magnification. Data shown are representative of 11 samples analyzed. **C.** MFI values for FITC fluorescence of goat anti-IgM and IgD pAbs and goat IgG pAbs of CLL BR Cohort 1, **D**. CLL BR Cohort 2, and **E**. normal B cells from healthy donor PBMCs. In all tested cases, there were no appreciable levels of non-specific binding of control species IgG on $CD19^+CD5^+$ and $CD19^+CD5^-$ cells. **F.** Representative measurement of median cell size. The mask adapts to the perimeter of the cell, and size is calculated based on the "area" feature that counts the total mask area in microns squared (μm^2). **G.** Representative measurement of high-density region / spot count and average area. Spot mask delineates associated pixels that comprise a bright region compared to background fluorescence. The feature "Spot Count" is then used to enumerate the number of regions identified. The average spot area is calculated based on the total "Spot mask" area divided by the number of spots. **H**. Comparison of isotype-specific MFI, spot count and spot area for $CD19^+CD5^-$ and $CD19^+CD5^+$ B cells from healthy donor PBMCs. Each dot represents the

median value of one sample. Bars represent group means ± SEM. **I**. Correlation of the DvM ratio with matching leukemic B-cell BRs for each patient. For statistical analyses, the following tests were applied: (**H**) One-Way ANOVA with Tukey test and (**I**) Pearson correlation coefficient.

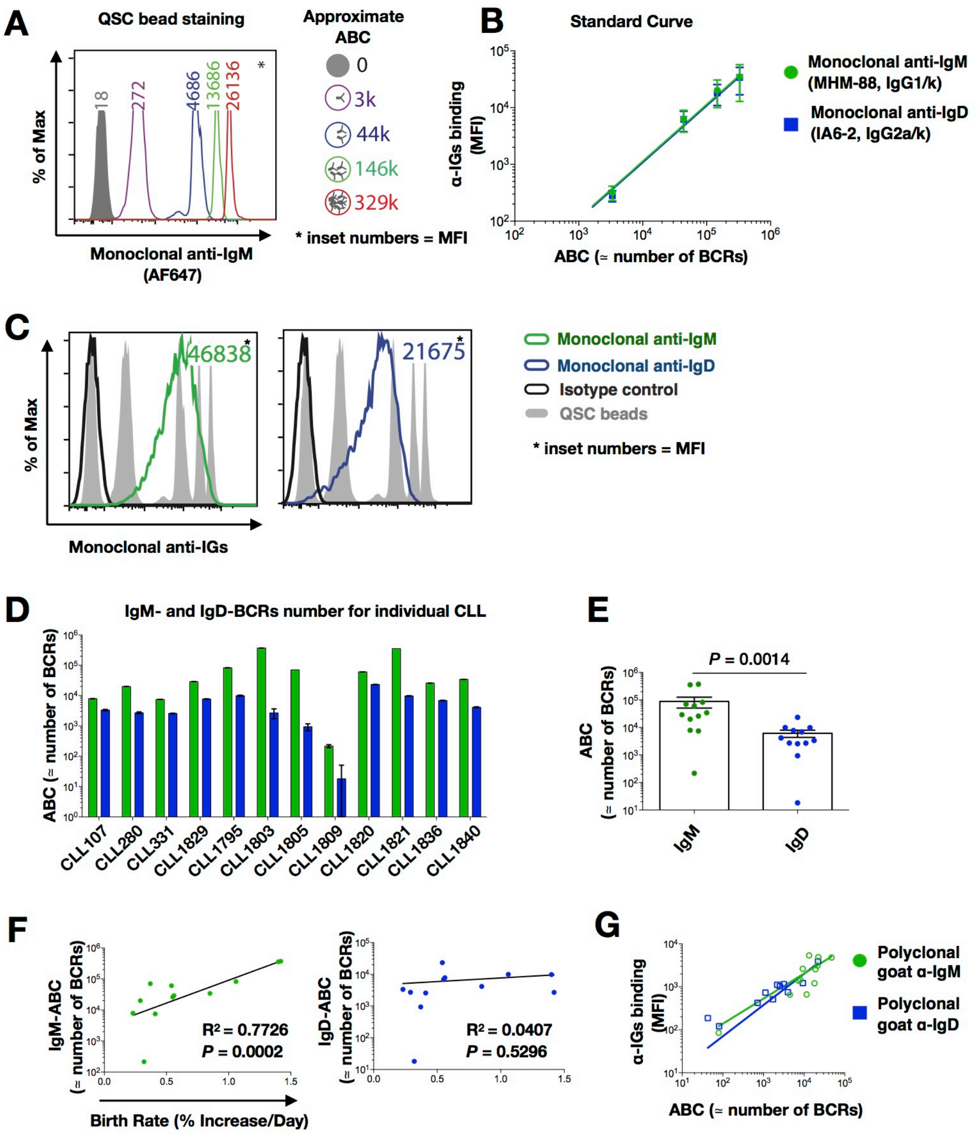

Figure S3. The absolute amount of membrane IgM and not IgD is linked with *in vivo* CLL birth rate. A. MFI values using monoclonal anti-IgM for Quantum Simply Cellular (QSC) beads with increasing Antigen Binding Capacity (ABC). **B**. Standard curve using QSC beads based on the double plot of MFIs determined by anti-IgM (green) and -IgD (blue) mAbs versus corresponding ABC. **C**. Representative staining of CLL sample with anti-IgM (green), anti–IgD (blue) mAbs and isotype control (Black) in comparison to isotype-matched QSC beads with different ABC (grey). **D**. Matched comparison of IgM (green) and IgD (blue) BCR numbers for

each individual sample. **E**. Pooled comparison of IgM (green) and IgD (blue) BCR numbers. Each dot represents the value of one sample. **F**. Correlation of IgM (green, left) and IgD (blue, right) number of BCRs on CLL B-cell membrane with matching B-cell birth rate (BR) measured for each patient as percent change in $CD19^{+}CD5^{+}$cells per day. Each dot represents the median value of one sample. **G**. IgM and IgD MFI of $CD19^{+}CD5^{+}$ cells upon normalization over their respective isotype control and plotted versus the known CLL B-cell ABC; this reveals a comparable linear translation for both isotypes. Each dot represents the MFI and ABC of a single patient. IgM and IgD slopes are equal ($P = 0.1394$). For statistical analyses, **(E)** the Mann-Whitney test was applied and, **(F)** Pearson correlation coefficient was used.

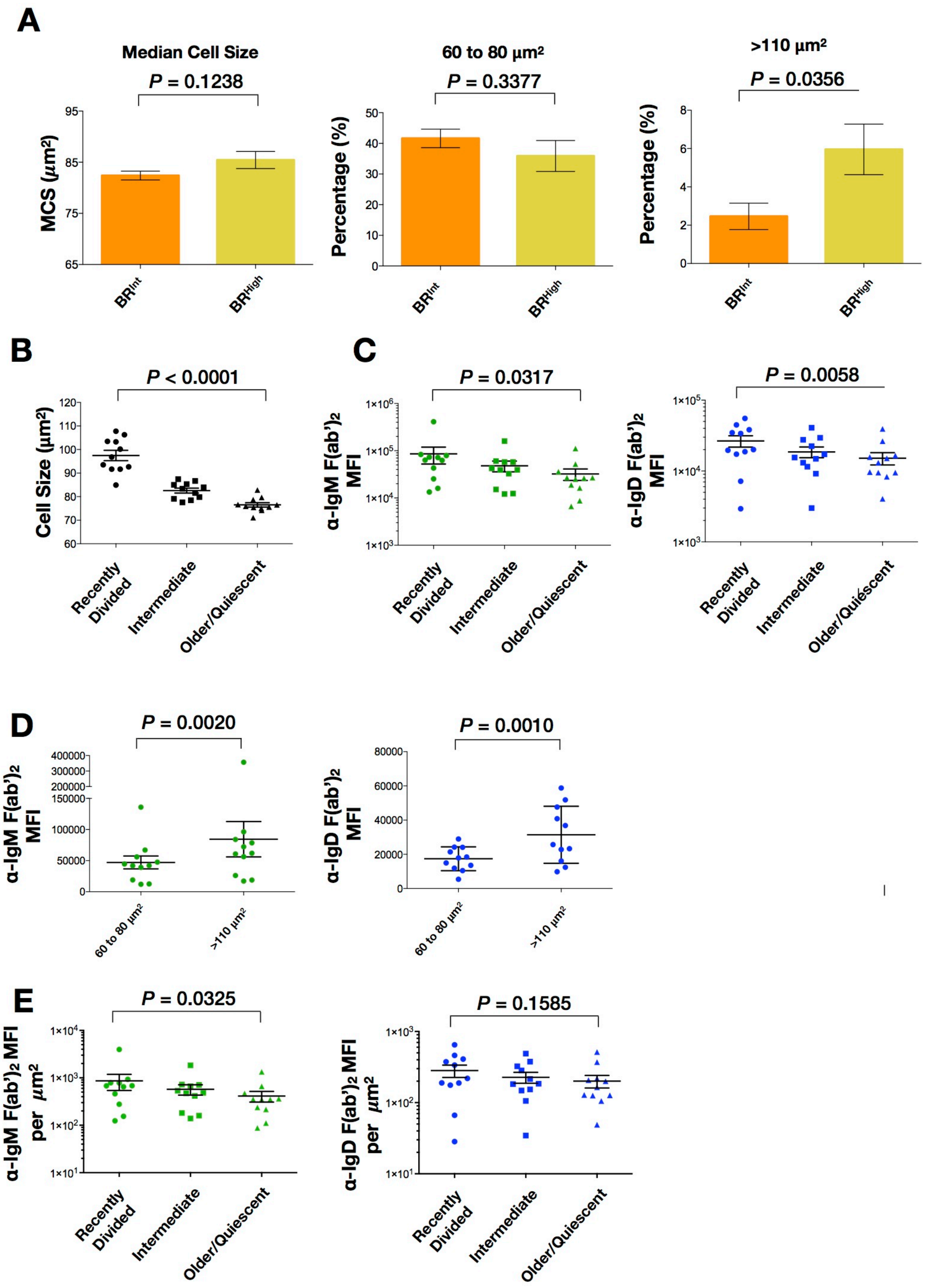

Figure S4. Cell size difference between CLL B cells with different *in vivo* birth rates and times since last cell division. A. Differences in MCS and percentage of smallest and largest CLL B-cell subpopulations for CLL BR 0.35% - 0.65% daily (n = 6, orange) and CLL BR 0.80% - 1.42% (n = 5, yellow). **B** and **C.** Analyses of cells differing in times since last division based on

the reciprocal expression of CXCR4 and CD5. Recently divided ($CXCR4^{Dim}CD5^{Bright}$), Intermediate ($CXCR4^{Int}CD5^{Int}$), and Older/Quiescent ($CXCR4^{Bright}CD5^{Dim}$) cell fractions are reported. **B.** Cell size measured in μm^2. **C.** Membrane levels of IgM (green) and IgD (blue) measured based on MFI. **D.** IgM (green) and IgD (blue) membrane levels in the smallest (60 - 80 μm^2) and largest (> 110 μm^2) cells. **E.** Analyses of cells differing in times since last division based on the reciprocal expression of CXCR4 and CD5. Recently divided ($CXCR4^{Dim}CD5^{Bright}$), Intermediate ($CXCR4^{Int}CD5^{Int}$), and Older/Quiescent ($CXCR4^{Bright}CD5^{Dim}$). IgM MFI (green) and IgD MFI (blue) were normalized to the average cellular membrane area of the corresponding subpopulation to calculate the IGs density per μm^2. For statistical analyses, the following tests were applied: (**A, D**) Mann-Whitney test and (**B, C, E**) One-Way ANOVA with Tukey test.

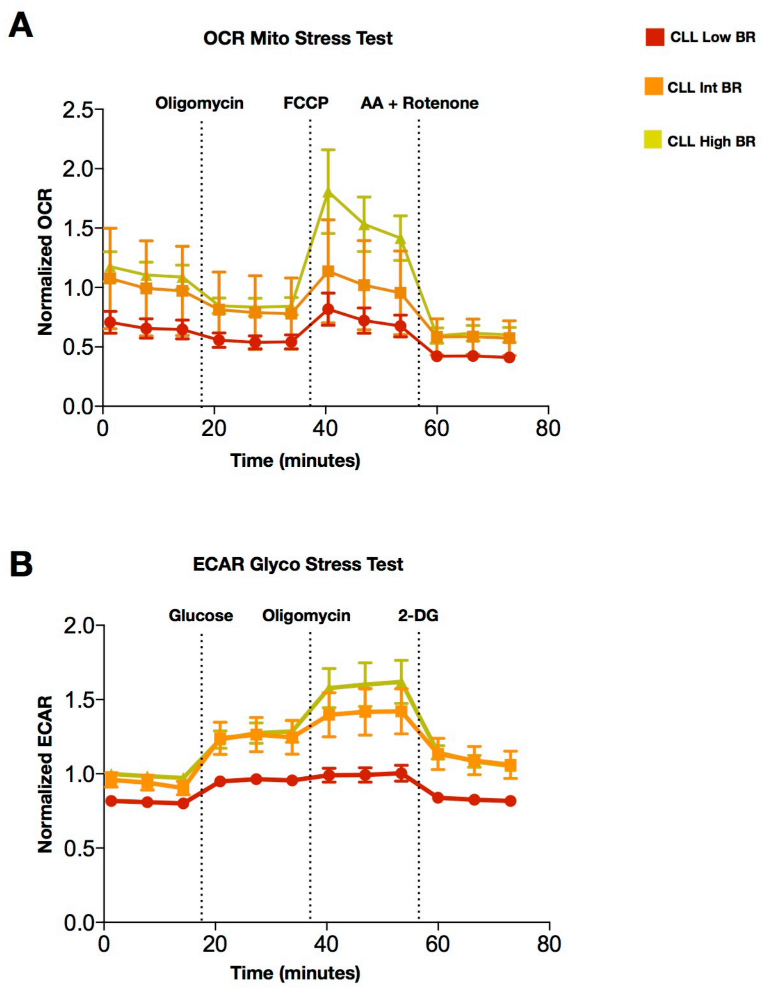

Figure S5. Oxygen Consumption Rate (OCR) and Extracellular Acidification Rate (ECAR) of CLL B cells differing in *in vivo* birth rates (BRs). A. OCR measurements for Mitochondrial Stress Test, and **B**) ECAR measurements for Glycolysis Stress Test. Each dot represents the average OCR and ECAR values for all patients within the CLL BR group ± SEM. CLL Low (n = 5; BR < 0.35%), CLL Int (n = 7; BR = 0.35 - 0.65%) and CLL High (n = 6; BR = 0.80 - 1.42%). OCR and ECAR were normalized defining 0% as the raw value of 0 and 100% as the last raw value of each dataset (or first, whichever was larger) and presented as fractions. Dashed lines separate values before or after each specific injection.

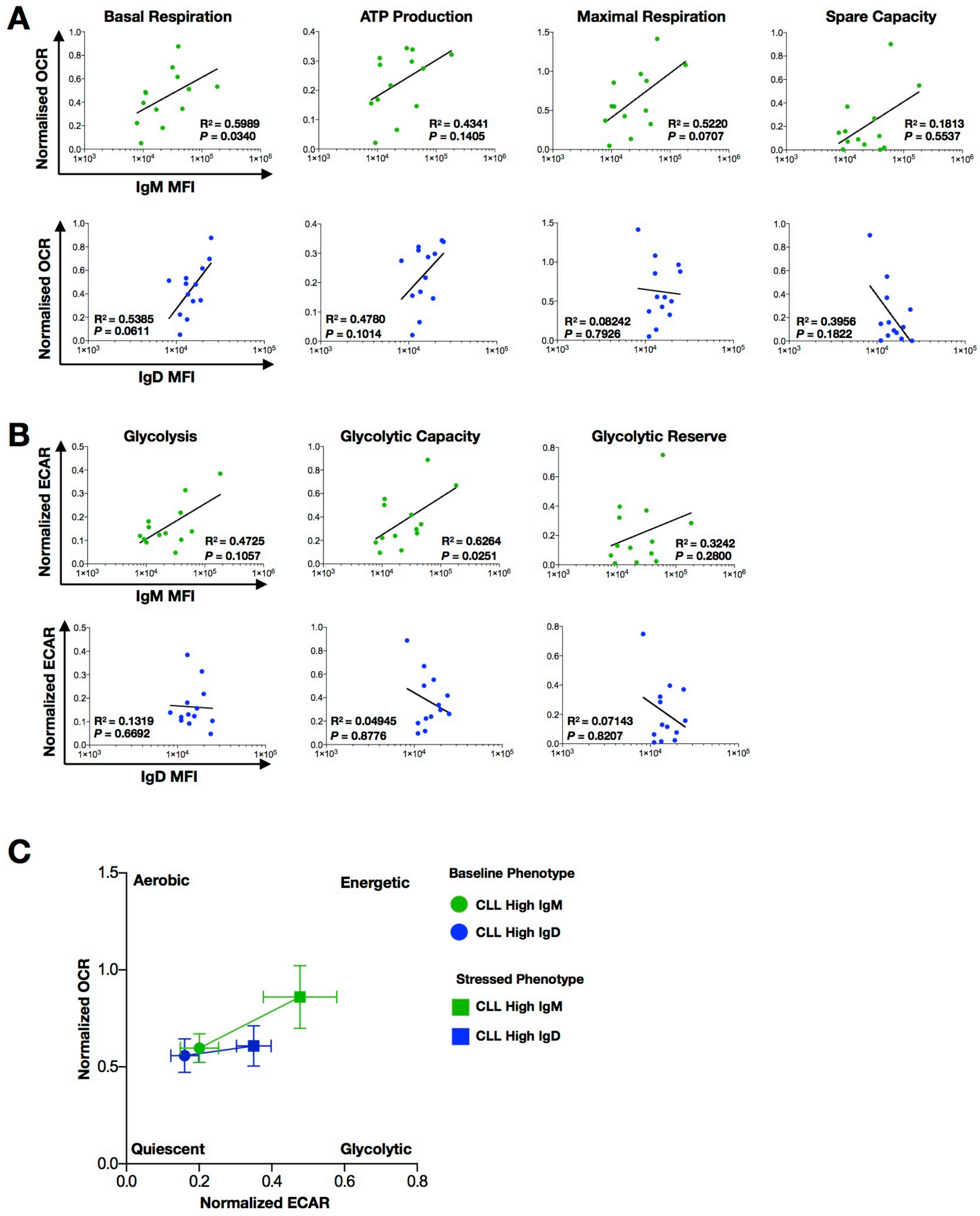

Figure S6. CLL-cell metabolic activity associates with CLL B-lymphocyte IgM and IgD MFI. **A**. Mitochondrial respiration parameters calculated based on Oxygen Consumption Rate (OCR) from Mitochondrial Stress Assay comparing CLL cases correlating OCR with matched IgM (top) and IgD (bottom) MFIs. **B**. Glycolytic parameters calculated based on Extracellular Acidification Rate (ECAR) from Glycolytic Stress Assay comparing CLLs correlating ECAR with matched IgM

(top) and IgD (bottom) MFIs. **C**. Metabolic profiles for CLL cases expressing high IgM or IgD MFI. Each dot represents the average OCR and ECAR values for all patients within the group ± SEM. Baseline Phenotype = Basal respiration (OCR) versus Glycolysis (ECAR); Stressed Phenotype = Maximal Respiration (OCR) versus Glycolytic Capacity (ECAR). High IGs CLL subgroups were defined as the 50% of cases with highest IgM or IgD MFI from the total population. OCR and ECAR were normalized defining 0% as the raw value of 0 and 100% as the last raw value of each dataset (or first, whichever was larger) and presented as fractions. The Pearson correlation coefficient was used for statistical analyses.

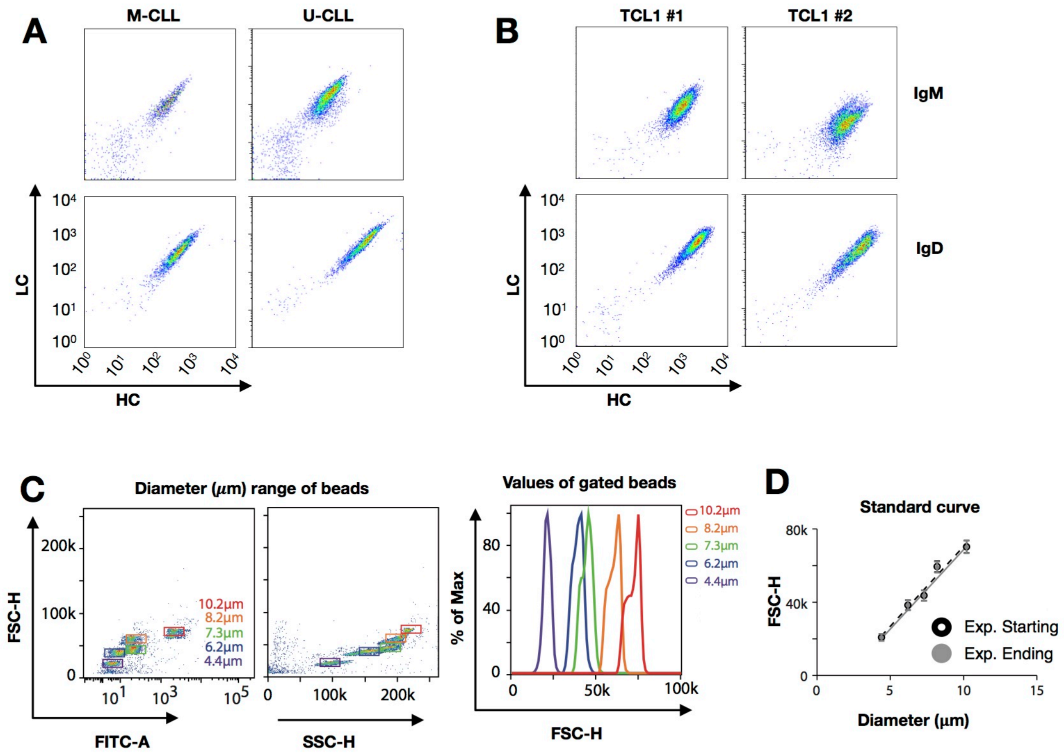

Figure S7. Membrane expression of IGs from CLL patients and TCL1 mice and TKO cell size measurement upon CLL BCR expression. A. Flow-cytometric analysis of IG Heavy chain (HC) and Light (LC) chain protein levels in TKO cells expressing the IgM and IgD versions of two representative CLL-BCRs. **B**. Similar analyses of two representative BCRs from TCL1 mice. **C**. Gating for size calibration standard (SCS) beads ranging from 4-11 µm diameter (left) and relative FCS-H values (right). **D**. Standard curve for SCS based on double plot of FCS-H versus matched diameter at the beginning (black) and end (grey) of experiment.

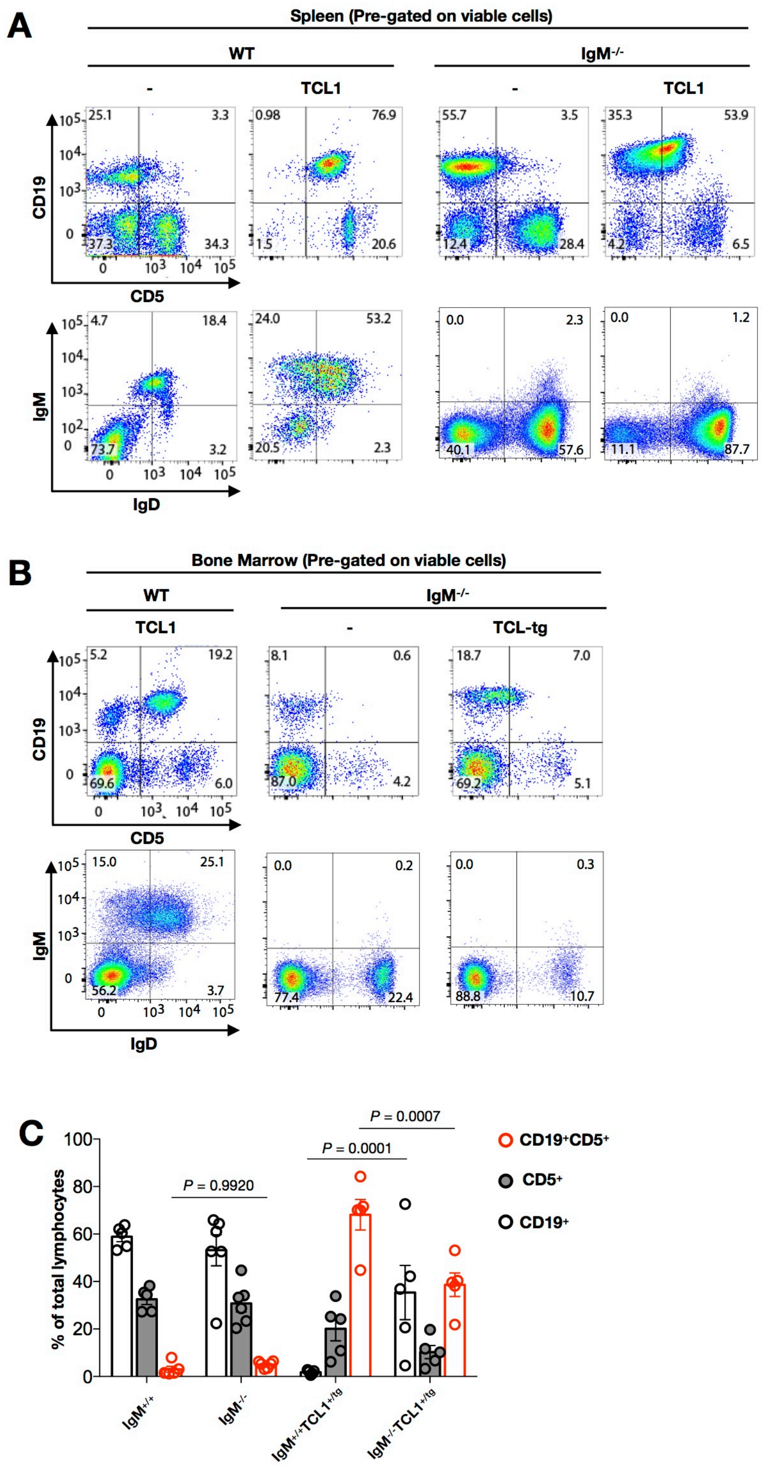

Figure S8. Phenotypic categorization of B lymphocytes in TCL1 mice. **A**. Flow-cytometric analyses of spleen cells. IgM$^{+/+}$C57BL6, IgM$^{+/+}$C57BL6-TCL1, IgM$^{-/-}$C57BL6, and IgM$^{-/-}$C57BL6-TCL1 mice are displayed (left to right) for expression of the indicated surface markers.

B. Flow-cytometric analyses of bone marrow cells. $IgM^{+/+}$C57BL6-TCL1, $IgM^{-/-}$C57BL6, and $IgM^{-/-}$C57BL6-TCL1 mice are displayed (left to right) for expression of the indicated surface markers. Numbers represent the percentage of cells in the respective quadrants. Mice were > 52 weeks old. **C**. Percentage of $CD19^+$, $CD5^+$ and $CD19^+CD5^+$ lymphocytes in spleens from $IgM^{+/+}$ (n = 5), $IgM^{-/-}$ (n = 6), $IgM^{+/+}$ TCL1 (n = 5) and $IgM^{-/-}$ TCL1 (n = 5) mice. Each point represents the subpopulation percentage of total splenic cells of one mouse of >12 months of age. Bar represents group means ± SEM. For statistical analyses, Two-Way ANOVA with Tukey test was used.

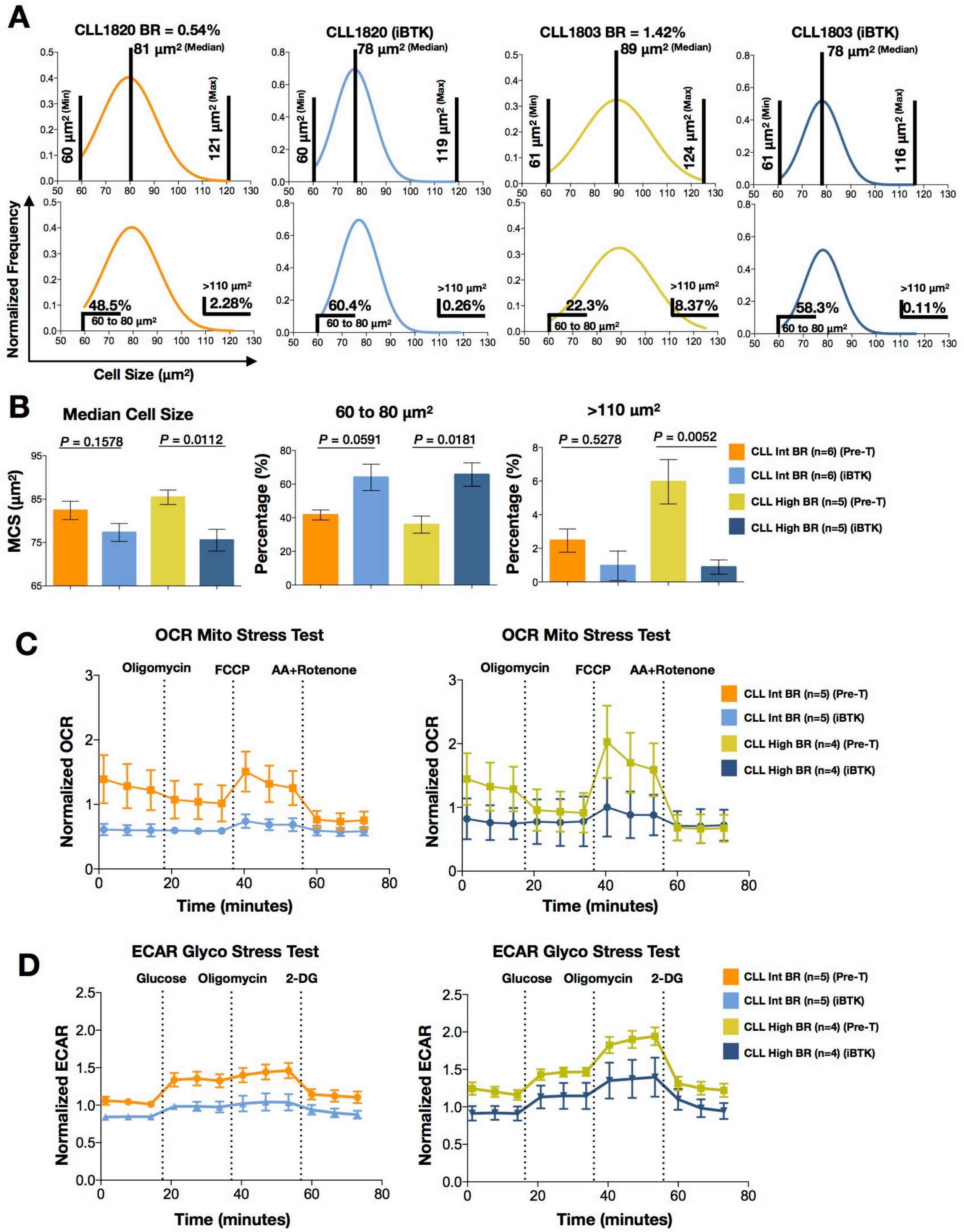

Figure S9. Effects of ibrutinib on cell size and metabolic activity of CLL B cells with different *in vivo* birth rates (BRs). A. Distribution of single cell areas for CLL1820 (BR 0.54% daily) before (Pre-T, orange) and during (light blue) ibrutinib treatment, and CLL1803 (BR 1.42%) before (Pre-T, yellow) and during (dark blue) ibrutinib treatment. Limits and medians of cell size

are shown. The same curves are duplicated at top and bottom to improve graphic display of data. **B.** Differences in MCS (left) and percentage of smallest (middle) and largest (right) CLL B-cell subpopulations for CLL Int BR group 0.35-0.65% (n = 6) and CLL High BR group 0.80 - 1.42% (n = 5), before (Pre-T) and during treatment. **C.** OCR and **D.** ECAR measurements before and during treatment with ibrutinib for the two groups based on BR. CLL Int BR 0.35 - 0.65% (n = 5) and CLL High BR 0.80 - 1.42% (n = 4). A One-Way ANOVA with Tukey test was used for statistical analysis.

Supplementary Tables

Table S1. Characteristics of the cohort of randomly selected CLL patients studied for IGHV mutation status, TTFT, and ZAP70 association with membrane IgM and IgD levels. IGHV mutational status (U, IGHV-unmutated CLL; and M, IGHV-mutated CLL); IgM and IgD Mean Fluorescence Intensity (MFI); IgD MFI and IgM MFI ratio (DvM), treatment required (Y/N) and; ZAP70 measured by immunohistochemistry (IHC).

Table S2. Characteristics of the two cohorts of CLL patients for whom clonal birth rates were measured *in vivo*. Birth rate (BR), % of newly born cells daily based on *in vivo* ^{2}H incorporation into newly synthesized DNA; IGHV mutational status (U, IGHV-unmutated CLL; and M,IGHV-mutated CLL); ZAP70 measured by immunohistochemistry (IHC); Fluorescence in situ hybridization (FISH); % of membrane $CD38^{+}$ cells within a CLL clone measured by flow cytometry; clinical response to ibrutinib treatment (PR = partial remission); and set of experiments

for which individual cases were employed (*) or not available (NA), imaging flow cytometry (IFC), extracellular acidification rate (ECAR) and oxygen consumption rate (OCR).

Table S3. Characteristics of the BCRs derived from human CLLs and TCL1 mice analyzed for autonomous and ligand-initiated BCR signaling using TKO cells.

Table S4. Frequency of IGHV use in splenic B cells of IgM$^{+/+}$TCL1, IgM$^{-/-}$TCL1, and IgM$^{-/-}$ mice. Mouse identification number (ID), genotype, IGHV used, and corresponding frequency within the splenic B cells are reported (left). Pie charts indicate IGHV used in IGHV-IGHD-IGHJ rearrangements for each genotype (right).
